# Optimization and application of non-native *Phragmites australis* transcriptome assemblies

**DOI:** 10.1371/journal.pone.0280354

**Published:** 2023-01-23

**Authors:** Feng Tao, Chuanzhu Fan, Yimin Liu, Subashini Sivakumar, Kurt P. Kowalski, Edward M. Golenberg

**Affiliations:** 1 Department of Biological Sciences, Wayne State University, Detroit, MI, United States of America; 2 U.S. Geological Survey-Great Lakes Science Center, Ann Arbor, MI, United States of America; Youngstown State University, UNITED STATES

## Abstract

*Phragmites australis* (common reed) has a cosmopolitan distribution and has been suggested as a model organism for the study of invasive plant species. In North America, the non-native subspecies (ssp. *australis*) is widely distributed across the contiguous 48 states in the United States and large parts of Canada. Even though millions of dollars are spent annually on *Phragmites* management, insufficient knowledge of *P*. *australis* impeded the efficiency of management. To solve this problem, transcriptomic information generated from multiple types of tissue could be a valuable resource for future studies. Here, we constructed forty-nine *P*. *australis* transcriptomes assemblies via different assembly tools and multiple parameter settings. The optimal transcriptome assembly for functional annotation and downstream analyses was selected among these transcriptome assemblies by comprehensive assessments. For a total of 422,589 transcripts assembled in this transcriptome assembly, 319,046 transcripts (75.5%) have at least one functional annotation. Within the transcriptome assembly, we further identified 1,495 transcripts showing tissue-specific expression pattern, 10,828 putative transcription factors, and 72,165 candidates for simple sequence repeats markers. The identification and analyses of predicted transcripts related to herbicide- and salinity-resistant genes were shown as two applications of the transcriptomic information to facilitate further research on *P*. *australis*. Transcriptome assembly and selection would be important for the transcriptome annotation. With this optimal transcriptome assembly and all relative information from downstream analyses, we have helped to establish foundations for future studies on the mechanisms underlying the invasiveness of non-native *P*. *australis* subspecies.

## Introduction

*Phragmites australis* (Cav.) Trin. Ex Steud., known as common reed, is a perennial, aquatic grass with highly variable phenotypic traits and a large range in ploidy level (3×, 4×, 6×, 7×, 8×, 10×, 11×, and 12×) [1, 2]. Because of its high adaptability to a range of ecosystems and ability to spread quickly by rhizome, stem node, or seed, *P*. *australis* has a cosmopolitan distribution and might be the most widespread plant species in the world [3]. Therefore, it makes *P*. *australis* one of the noxious invasive species in the world. The non-native subspecies of *P*. *australis* (ssp. *australis*) increased its spread to all 48 of the contiguous United States and large parts of Canada over the past 150 years [4]. Dense, monoculture stands caused by the non-native subspecies of *P*. *australis* often compete and replace the diverse native subspecies (ssp. *americanus*) communities [5]. In response to this rapid native community destruction, state and provincial natural resources agencies implemented *Phragmites* management regimens that included flooding, mowing, burning, and herbicide treatments, all at significant costs. From 2005 to 2009, approximately $4 million annually was spent on *Phragmites* management in the US; however, some efforts seemed ineffective and yielded little ecological benefit [6–8]. Given the impact of this species, Meyerson, Cronin [9] suggested using *P*. *australis* as a model species for studying plant invasions. As such, further research on this species is required, including transcriptomic studies on multiple types of tissues [10].

Ten years ago, the RNA sequencing (RNA-seq) approach was predicted to “revolutionize the manner in which eukaryotic transcriptomes are analyzed” [11]. Since that time, the RNA-seq approach has been used in many different fields. In ecology studies, most of the samples used in RNA-seq are from non-model organisms. The expression differences underlying interindividual or interpopulation variation in ecologically important traits could still be examined without the need for prior genomic reference [12]. Such studies have been directed to *Phragmites* as well [1, 3, 13–15]; however, to our knowledge, most of the works focus on either identifying salinity-response genes or distinguishing differentially expressed genes between tetraploid and octoploid plants. Moreover, all of these studies only used RNA-seq data from one or two types of tissue (leaf and/or rhizome) to perform *de novo* transcriptome assembly, and the samples analyzed were from plants grown in controlled, experimental conditions. Considering that every type of tissue has a specific expression pattern, the transcriptome generated from one or two types of tissue might not fully represent the gene expression patterns in the plant. Additionally, most of those previous studies only used one *de novo* assembly tool (commonly Trinity) and did not examine the transcriptome with sufficient quality assessments. Practically, studies have already evaluated several *de novo* transcriptome assembly tools and parameter settings and found deficiencies in transcriptome generated only from one assembly tool and with one parameter setting [16–18]. As an alternative, the generation of multiple transcriptome assemblies by different assembly tools and parameter settings, and collating them as one non-redundant transcriptome for a more robust and reliable result have been suggested [17–20]. Meanwhile, selection in each step of *de novo* transcriptome assembly can also affect the quality of the transcriptome [17]. As a result, to construct a high-quality *P*. *australis* transcriptome, strategies such as using multiple types of tissues, constructing transcriptome assemblies via several bioinformatics tools and parameter settings, and comprehensive quality assessments should be considered.

**Fig 1:**
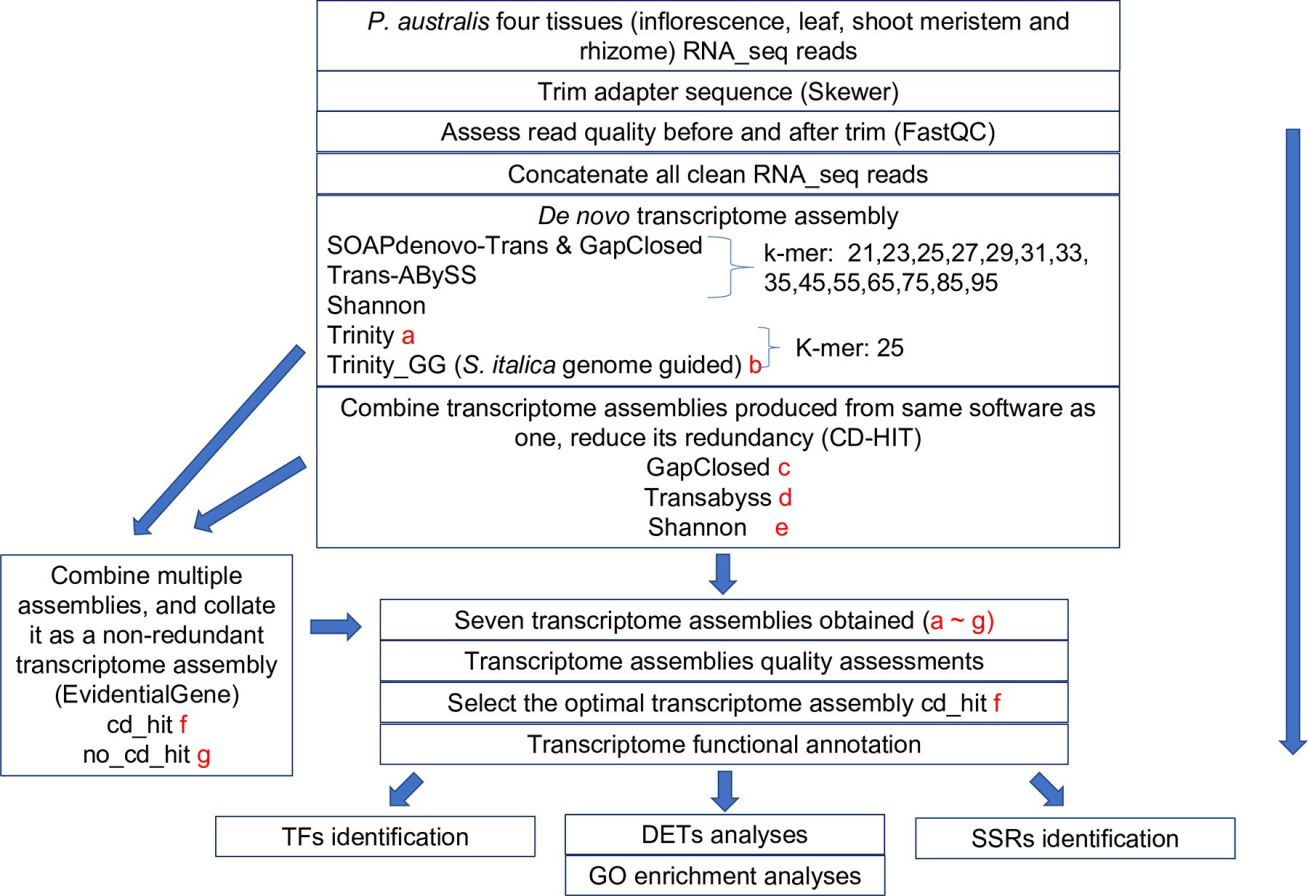
Experiment flowchart of transcriptome assembly and downstream analyses. Red color letters (a to g) are the selected seven transcriptome assemblies for quality assessments from forty-nine de novo assembly transcriptome assemblies.

In this study, RNA-seq data was generated via using multiple types of tissue from a non-native *P*. *australis* plant that was grown in an open field of urban Detroit, Michigan, US. This location in the Laurentian Great Lakes region is severely affected by non-native *P*. *australis* subspecies (ssp. *australis*) [4]. We constructed forty-nine transcriptome assemblies via different assembly tools and parameter settings, selected the optimal transcriptome assembly with careful quality assessments, and comprehensively annotated its function. To understand *P*. *australis* better through the transcriptomic data, three downstream analyses were performed: (1) identification of differentially expressed transcripts (DETs) among tissues followed by Gene Ontology (GO) terms enrichment analyses, (2) identification of putative transcription factors (TFs), and (3) detecting candidate transcripts with simple sequence repeats (SSRs) markers. The flowchart for the whole analysis is shown in Fig 1. Through these findings, we aim to build a valuable resource for the non-native *P*. *australis* subspecies, including an optimal transcriptome assembly and relative information. As two applications of this resource, we identified predicted transcripts with herbicide- and salinity-resistant functions and viewed their expression patterns among tissues. This resource may serve as a guide for future *P*. *australis* studies.

## Materials and methods

### Plant material

The non-native *P*. *australis* plant material used for the experiment was sampled from an open field in urban Detroit, Michigan, US (42.326919, -83.077062) in March 2013 with permission from Ford Motor Company, the owner of the sample site property. The identification of the plant specimen was independently confirmed by Dr. Donna Kashian who is a professor and ecologist/botanist in the Department of Biological Sciences, Wayne State University. The voucher for the specimen was placed in the Wayne State University herbarium (Accession number 2902). Four types of plant tissue were collected including inflorescence, leaf, shoot meristem protruding from the rhizome, and rhizome. Tissues were washed in distilled water to remove surface soil, placed in sterile 50mL plastic tubes, and stored immediately in dry ice. The samples were transferred to the -80 freezer in the laboratory and later separately grounded to the powder in mortars and pestles with liquid nitrogen.

### RNA isolation and sequencing

Total RNA was extracted using Direct-zol™ RNA MiniPrep with TRIzol®. DNA was removed by DNase I digestion and added to the extraction column following the manufacturer’s protocol. RNA concentrations and qualities were estimated from 260/280 absorbance ratios via using a Nanodrop spectrophotometer. Due to the cost of sequencing at the time (2013), samples did not have any biological and technical replicates. Paired-end RNA-seq library for each tissue was made using the ScriptSeq kit (Epicentre) following the manufacturer’s protocols with minor modifications. 5µg of RNA were treated with Ribo-zero beads (Seed/Root beads are specific for rhizome and shoot meristem derived RNA; Leaf beads are specific for leaf and inflorescence derived RNA) to deplete ribosomal RNA. Approximately 50ng of rRNA-depleted RNA from each tissue source was fragmented according to protocol with the exception that fragmentation at 85°C was reduced from the recommended 5 minutes to 30 seconds. First-strand cDNA was made using random hexamers. This cDNA was terminally tagged and then amplified for 17 cycles using barcoded primers for each library based on tissue of origin. In the end, sequences were determined on an Illumina HiSeq 2000 by RTSF genomics core at Michigan State University.

### Read processing and *de novo* transcriptome assembly

First, adapters from the raw reads were trimmed and low-quality reads were filtered out using Skewer-0.22 [21]. The quality of raw and after-trimmed sequences was checked using FastQC v0.11.8 [22] and the Phred score of trimmed reads was estimated by q30-master. These high quality and adapter-free reads from four tissues were merged as two files and used for *de novo* transcriptome assembly. Five different strategies using different assembly tools and parameter settings were applied: 1. We used Trinity-2.8.4 [23] with the default kmer 25 to assemble the transcriptome and named it “Trinity” (transcriptome assembly a); 2. We applied another Trinity script to perform genome-guided transcriptome assembly with the default kmer 25. As there was no reference genome of *P*. *australis* when this project was started, this analysis was based on the *S*. *italica* v2.2 genome sequence from Joint Genome Institute [24]. Following the instruction, we first generated the bam file by aligning two merged read files to the *S*. *italica* genome via STAR-2.5.2b [25]. A transcriptome assembly could then be obtained by using this bam file and was named as “Trinity_GG” (transcriptome assembly b); 3. Fourteen transcriptome assemblies were generated via Shannon-0.02 [26] with fourteen kmer settings: 21, 23, 25, 27, 29, 31, 33, 35, 45, 55, 65, 75, 85, 95; 4. We then generated another fourteen transcriptome assemblies via SOAPdenovo-Trans-v1.03 [27] with the same fourteen kmer settings and reduced the size of the gaps of these fourteen transcriptome assemblies with GapCloser-1.12 after; 5. We obtained the third set of fourteen transcriptome assemblies via Trans-ABySS-2.0.1 [28] with the same fourteen kmer settings.

To collate multiple assemblies from the previous step to one non-redundant transcriptome assembly, two methods were used. First, for the last three non-Trinity strategies [3–5], we concatenated fourteen assembly files which were generated via different kmer settings but from each strategy. The redundancy at nucleotide identity of 98% was reduced for three combined files via CD-HIT-EST from CD-HIT-4.8.1 [29]. Each redundant reduced file was named “GapClosed”, “Transabyss”, and “Shannon”. They were referred to as transcriptome assembly c, d, and e, respectively. We then combined them with transcriptome assembly a and b as one file. This combined file was processed by the tr2aacds pipeline from EvidentialGene17dec14 [30] to generate one transcriptome assembly, which was named “cd_hit” (transcriptome assembly f); Second, unlike the first method, all the forty-four transcriptome assemblies were combined as one file without the procedure for eliminating redundancy by CD-HIT-4.8.1. We processed this combined file through the tr2aacds pipeline directly and named this transcriptome assembly “no_cd_hit” (transcriptome assembly g).

### Transcriptome assemblies’ quality assessments

To assess and compare the quality of seven transcriptome assemblies a~g, corresponding to “Trinity”, “Trinity-GG”, “Shannon”, “Transabyss”, “GapClosed”, “cd-hit”, and “non-cd-hit”, respectively, several assessment tools were utilized: 1. Benchmarking universal single-copy orthologs (BUSCO) 4.0.5 [31] was first used to test the completeness of these seven transcriptome assemblies. Two different lineage datasets poales_odb10 and embryophyte_odb10 were downloaded from the BUSCO library and used separately; 2. The traditional method N50 and modified method EX90N50 were also calculated for the comparison. As the size of many lowly expressed transcripts are normally small and might bias the N50 value, N50 among the topmost highly expressed genes could avoid this bias. Therefore, taking transcripts that represent 90% of the total normalized expression data is suggested (known as EX90N50) [32]. N50 value was calculated by the Trinity script TrinityStats.pl, and Ex90N50 was obtained via the other Trinity script contig_ExN50_statistic.pl; 3. Using Blastx from ncbi-blast-2.8.1+ [33] and Trinity package script analyze_blastPlus_topHit_coverage.pl, we searched each transcriptome assembly against the UniProt-Swissprot database [34] to count the representation of nearly full-length (> 80% alignment coverage) constructed protein-coding genes using an e-value 10^−20^ and single best match. Meanwhile, we applied the same strategy to align each assembled transcriptome to the annotated transcriptome of *S*. *italica* v2.2 via Blastn and counted the representation of nearly full-length constructed *S*. *italica* genes; 4. We used two sets of *P*. *australis* RNA-seq data to examine the mapping rate to each transcriptome assembly via bowtie2-2.3.0 [35]. The first set was the combined reads from our study. The second set was downloaded from PRJNA314710. These data were from Australian *P*. *australis*, which was extracted from low salinity sites and grown in freshwater before sequencing [14]. The same procedure of Skewer was used to treat this data set as well; 5. To compute DETONATE scores and generate some basic transcripts metrics such as transcripts length distribution, we tried the other two software tools DETONATE-1.11 [36] and rnaQUAST v2.2.0 [37].

### Transcriptome functional annotation

Before the application of the Trinotate-v.3.2.1 pipeline [38] for transcriptome assembly functional annotation, we used the TransDecoder-v5.5.0 pipeline [39] to extract and predict the likely protein coding regions within transcripts from the transcriptome assembly “cd_hit” that was selected after the transcriptome assembly quality assessments. This predicted peptide sequence file (amino acid sequences) and the original transcriptome assembly file (nucleotide sequences) were separately applied for the transcriptome functional annotation against the chosen databases. To follow the Trinotate pipeline [40], we carried out the following analyses: 1. We aligned both files against the UniProt-Swissprot protein database using tools from ncbi-blast-2.8.1+; 2. We used both files via the same tool as analysis 1 against *S*. *italica* v2.2 annotated protein file; 3. Considering the large volume of RefSeq non-redundant proteins database (NR) [41] and clusters of orthologous genes database (COG) [42], we aligned both files against these two large databases via DIAMOND v2.0.6 [43] to shorten the processing time; 4. To find the protein domain, we used the predicted peptide sequence file to search against the Pfam v33 database [44] via using hmmer-3.3 [45]. All the e-value cut off used for the above alignments were 10^−3^ and the best match was chosen; 5. The predicted peptide sequence file was also used in other methods based on the Trinotate pipeline. They included using SignalP-4.1 [46] for searching signal peptides and using tmhmm-2.0c [47] for searching transcripts with transmembrane regions. GO terms were assigned from the results from the homology search against the Uniprot-Swissprot database. All these annotations were loaded into a Trinotate SQLite database; 6. The Kyoto Encyclopedia of Genes and Genomes (KEGG) term was also assigned from GhostKOALA [48] result via using the predicted peptide sequence file. In the end, annotations from the Trinotate SQLite database and KEGG terms were combined as the final report and outputted as a CSV file.

### DETs analyses

RNA-seq by expectation-maximization (RSEM) v1.2.19 [49] with bowtie2 [35] as the alignment tool was first used to align trimmed data from each tissue separately to the transcriptome assembly “cd-hit” and estimate its transcript-level read counts abundance. Trinity tools for differential expression analyses were successively used as: built transcripts expression metrics (abundance_estimates_to_matrix.pl), examined the correlation among samples (PtR), identified DETs in each pairwise comparison among samples (run_DE_analysis.pl with EdgeR Bioconductor package [50], extracted and clustered DETs (analyze_diff_expr.pl), and found main clusters based on expression pattern of transcripts (define_clusters_by_cutting_tree.pl). Since samples in our study do not have any replicates, we set the dispersion parameter as 0.1. The threshold FDR to extract DETs is < 0.005, and the fold change is ≥2. Venn diagrams to present up- and down-regulated transcripts from each pairwise comparison among tissues were generated from R package ggvenn. Lists of DETs showing tissue-specific expression patterns were also generated from the results of Venn diagrams.

The GO enrichment analysis for DETs in each pairwise comparison among samples could be directly performed via the Trinity script analyze_diff_expr.pl with the—examine_GO_enrichment parameter. The background for this set of analyses is the list of transcripts that show expression in all samples and have GO annotations. For GO enrichment analyses on transcripts that are differentially expressed in a specific tissue, we execute Trinity script run-GOseq.pl. The background data are the list of transcripts that show expression in that certain tissue and have GO annotations.

### Identification of putative TFs

We used the file of *S*. *italica* transcription factors (TFs) information as the reference, which was downloaded from PlantTFDB v5.0 [51]. We identified all putative TFs by comparing this reference with the transcriptome assembly “cd_hit” functional annotation file, in which we had the results from the alignment between the predicted peptide sequence file and *S*. *italica* annotated protein file via Blastp (e-value < 10^−3^ and best match selected). We screened TFs with tissue-specific expression patterns, based on the lists of tissue-specific DETs from the previous step.

### Candidate SSRs identification

MISA v2.1 [52] was applied locally to identify SSRs from the transcriptome assembly “cd-hit” with the default parameter settings as a minimum of 10 repeats for mononucleotide, 6 repeats for dinucleotide, 5 repeats for trinucleotide, tetra, penta, and hexanucleotide with a maximum interruption of 100 bases between two SSRs. Based on the lists of tissue-specific DETs from the previous step, we also searched transcripts having SSRs markers and showing tissue-specific expression patterns. We further selected the Coding sequence SSRs (CDS-SSRs) from this type of transcript by checking whether the SSRs are in the predicted protein coding region via the protein coding region file.

### Predicted herbicide- and salinity-resistant transcripts analyses

The transcripts within the transcriptome assembly “cd_hit” were defined as putative herbicide- or salinity-resistant genes based on the transcriptome functional annotation. Results for herbicide-resistant genes were from the Pfam annotation. If a transcript had cytochrome P450, glycosyltransferase (GT), or glutathione S-transferase (GST) domain, it was listed. Results for salinity-resistant genes were from the Uniprot-Swissprot annotation. If a transcript matched with Uniprot ID as *NHX7* (Q9LKW9), *CIPK24* (Q68Q47), *CBL4* (Q74KU4), *PK* (Q8W1X2), *FLA4* (Q9SNC2), or *SALT* (A2WPN7) (S3 Table in S1 File), it was counted. After obtaining these two lists of transcripts, we then checked their expression pattern based on the expression matrix and the lists of tissue-specific DETs obtained from previous analyses.

### Ethics approval and consent to participate

All methods complied with relevant institutional, national, and international legislation. Ford Motor Company permitted us to sample the plant material from their property.

## Results

### Sequencing and forty-nine transcriptome assemblies generated from *de novo* transcriptome assembly

For four types of tissue tested in this study, each tissue was used to build one paired-end library for sequencing (PF: inflorescence, PL: leaf, PM: shoot meristem, PR: rhizome). A total of 264 million reads of 100 bp RNA-Seq raw data were obtained, ranging from 58,171,634 to 84,369,782. After adapters and low-quality reads were removed by the gentle quality trimming tool Skewer, the number of clean reads from each sample is from 39,079,888 to 76,934,742 and all trimmed reads show high quality (at least 92% bases with Phred score above 30 for each sample) (Table 1).

**Table 1:** Statistics of raw and trimmed reads from *P*. *australis* four tissues RNA-Seq data.

Sample	Label	Amount of raw read	Amount of after-trimmed read	Kept percentage after trim	%> = Q30 after trim
Inflorescence	PF	83,450,674	76,934,742	91.2%	95.2%
Leaf	PL	84,369,782	76,382,488	91.5%	94.8%
Shoot meristem	PM	58,245,428	39,079,888	67.1%	94%
Rhizome	PR	58,171,634	47,632,556	81.9%	94%

Considering the limitations of *de novo* transcriptome assembly using a single software tool and with one kmer setting [17, 18, 20], seven transcriptome assemblies (a. “Trinity”, b. “Trinity_GG”, c. “GapClosed”, d. “Transabyss”, e. “Shannon”, f. “cd_hit”, and g. “no_cd_hit”) were chosen from forty-nine transcriptome assemblies for quality assessments (Fig 1). The number of transcripts of each transcriptome assembly varies, from 56,217 (“Trinity_GG”) to 1,653,659 (“GapClosed”), but the remaining five transcriptome assemblies are in the range of 400,000 to 700,000 (Table 2).

**Table 2:** Statistics of main transcriptome assembly assessments for seven constructed transcriptome assemblies.

	BUSCOs Poales*^a^	BUSCOs Embryop-hyte*^a^	N50 (bp)	EX90N50 (bp)	UniProt-Swissprot database coverage*^b^	*S*. *italica* transcriptome coverage*^b^	Total Transcripts
Trinity	3,276	1,088	684	1,741	7,277	5,264	506,626
Trinity_GG	886	276	584	1,282	2,013	533	56,217
GapClosed	2,726	892	286	1,269	6,178	4,437	1,653,659
Transabyss	3,362	1,243	596	1,703	8,461	6,589	673,152
Shannon	3,635	1,100	1,426	1,905	8,025	6,478	708,788
cd_hit	3,716	1,268	1,219	2,011	9,160	7,113	422,589
no_cd_hit	3,717	1,276	1,262	1,908	9,347	7,172	594,388

*^a^ Number of complete BUSCOs including single-copy and duplicated.

*^b^ 80% alignment coverage of UniProt-Swissprot database protein sequence or *S*. *italica* reference transcriptome genes with e-value less than 10^−20^.

### Quality assessments to select the optimal transcriptome assembly

**Fig 2:**
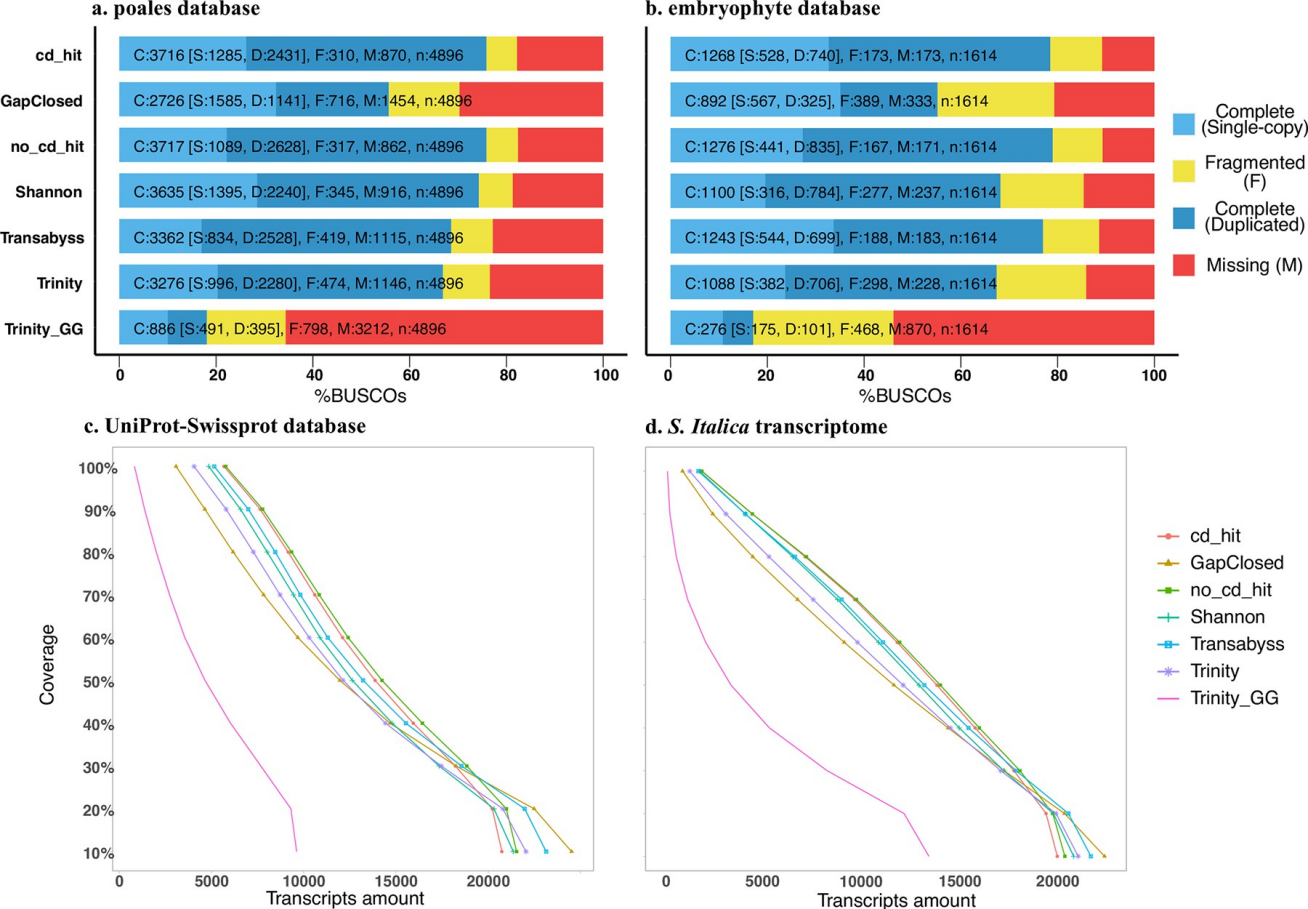
Quality assessments for seven constructed transcriptome assemblies. a and b. cumulative percentage of orthologues identified from the BUSCO search from two databases a. poales (n: 4,896) and b. embryophytes (n: 1,614) against the seven constructed transcriptome assemblies. Complete orthologue (C) can be either single-copy (S) or duplicated (D). Incomplete orthologues are considered fragmented (F). If orthologues from the database are not identified, they are marked as missing (M); c and d. the cumulative number of transcripts along with alignment coverage changes to annotated genes from two databases c. Uniprot-Swissprot and d. *S*. *italica* v2.2 transcriptome sequence file against the seven constructed transcriptome assemblies.

Without quality assessments, results of downstream analyses using the *de novo* assembled transcriptome might be distorted; therefore, several tools and evaluation methods are designed for this purpose [23, 31, 36, 37]. To select the optimal transcriptome assembly among the seven transcriptome assemblies, we performed comprehensive assessments. Among them, the first tool we used was BUSCO with two lineage datasets, embryophyte_odb10 and poales_odb10. Though transcriptome assembly “cd_hit” and “no_cd_hit” that are both processed by the tr2aacds pipeline have the most complete BUSCO matches (Table 2), the composition of complete BUSCO matches is not similar (Fig 2A and 2B). Transcriptome assembly “cd_hit” has more single-copy matches than transcriptome assembly “no_cd_hit” (embryophyte_odb10: 528 vs. 441; poales_odb10: 1285 vs. 1089), which might be due to the extra procedure to reduce redundancy via CD-HIT before tr2aacds pipeline. In contrast, transcriptome assembly “Trinity_GG” has the lowest number of complete BUSCO matches (Table 2). The procedure used to construct this transcriptome assembly might be responsible for it, as this transcriptome assembly was based on the *P*. *australis* closely relative species *Setaria italica* genome sequence and it might underestimate the contigs due to their genome sequence divergence.

The traditional method N50 and modified method EX90N50 were also calculated for the transcriptome assembly comparison. After calculation and comparison, transcriptome assembly “cd_hit” and “no_cd_hit” are both found in the top 3 of two statistics. The other transcriptome assembly in the top 3 of the two statistics is transcriptome assembly “Shannon” (Table 2).

We then counted the number of assembled transcripts that show nearly full-length matched (> 80% alignment coverage) with the gene sequence from the reference database (Table 2). Two databases used for this type of assessment were the UniProt-Swissprot protein sequence and *S*. *italica* v2.2 transcriptome sequence. We then compared the distribution of transcripts with different coverage for all the seven transcriptome assemblies and found that both examinations show a similar pattern (Fig 2C and 2D). Transcriptome assembly “cd_hit” and “no_cd_hit” have more nearly full-length matched transcripts; however, along with the decrease of the alignment coverage, the rank of both transcriptome assemblies in the two lists drops. When the alignment coverage is at >20%, the other four transcriptome assemblies “GapClosed", “Shannon”, “Transabyss”, and “Trinity” have more counts of matched transcripts. This result is consistent with the result from two BUSCO analyses, showing that these four transcriptome assemblies have more short fragments than transcriptome assembly “cd_hit” and “no_cd_hit”. These observations suggest that these kinds of fragments could not be integrated as a complete gene sequence, which was only processed by these name corresponding assembly tools.

Results from the remaining transcriptome assembly quality assessments performed in this study are in S1 Table in S1 File. Either using RNA-seq data generated from this project or the Australian *P*. *australis* project [14], all transcriptome assemblies have a mapping rate greater than 89% except transcriptome assembly “Trinity_GG”. The length distribution of transcripts of each transcriptome assembly is shown in S1 Fig, in which we could observe variances among these seven transcriptome assemblies. Similar phenomena also exist in all other statistics such as numbers of transcripts length > 500 bp, numbers of transcripts length > 1000 bp, maximum transcript length, and DETONATE score. All the above results demonstrate that *de novo* transcriptome assembly via one software and with one default kmer setting might not be the optimal choice, especially without comprehensive quality assessments.

In this study, we consider the transcriptome assembly with the most biological representative as the optimal one. Therefore, transcriptome assembly “cd_hit” and “no_cd_hit” are the best two candidates. Though both transcriptome assemblies show similar qualities in most of the assessments, transcriptome assembly “cd_hit” has 171,799 fewer transcripts than “no_cd_hit” (Table 2: 422,589 vs. 594,388). Since more redundant transcripts might bias the downstream analyses, especially in the differentially expressed transcripts analysis, transcriptome assembly “cd_hit” was selected as the optimal one among seven compared transcriptome assemblies for the downstream studies in the end.

### Candidate coding regions identification and transcriptome functional annotation for downstream analyses

To annotate the transcriptome assembly “cd_hit”, TransDecoder was firstly used to predict and identify the candidate coding regions within the transcripts. 229,640 transcripts are found to have coding regions with at least 100 aa in length (S2 Fig). Among them, 80,243 transcripts have complete CDS, 67,675 transcripts have 5 prime partial CDS, 25,853 have 3 prime partial CDS, and 55,869 have internal CDS.

To understand the transcriptome better, thirteen strategies involving six databases were applied for the transcriptome functional annotation (Table 3). The databases included those used for the Trinotate default annotation (UniProt-Swissprot protein database and Pfam database) and other frequently used databases (*e*.*g*., NR database, COG database, and KEGG database). Besides these databases, results generated from the *S*. *italica* v2.2 annotated protein file were added as well. The number of annotated transcripts by each strategy ranges from 10,802 (2.56% of all transcripts are predicted as putative signal peptides) to 304,567 (72.07% are matched in the NR database). Overall, 319,046 (75.50%) transcripts have at least one type of annotation.

**Table 3:** Summary for annotation results of transcriptome assembly “cd_hit”.

Type of annotation	Number of transcripts annotated	Percentage
Uniprot blastx*^1^	216,713	51.28%
Uniprot blastp*^2^	155,049	36.69%
*S*. *italica* blastx	234,500	55.49%
*S*. *italica* blastp	164,269	38.87%
NR blastx	304,567	72.07%
NR blastp	197,768	46.80%
COG blastx	83,637	19.79%
COG blastp	61,985	14.967%
Pfam	136,655	32.34%
Singalp	10,802	2.56%
tmhmm	30,274	7.16%
GO	87,165	20.60%
KEGG	91,545	21.66%
Total	319,046	75.50%

*^1^ blastx means using the transcriptome file

*^2^ blastp means using the candidate protein coding file

**Fig 3:**
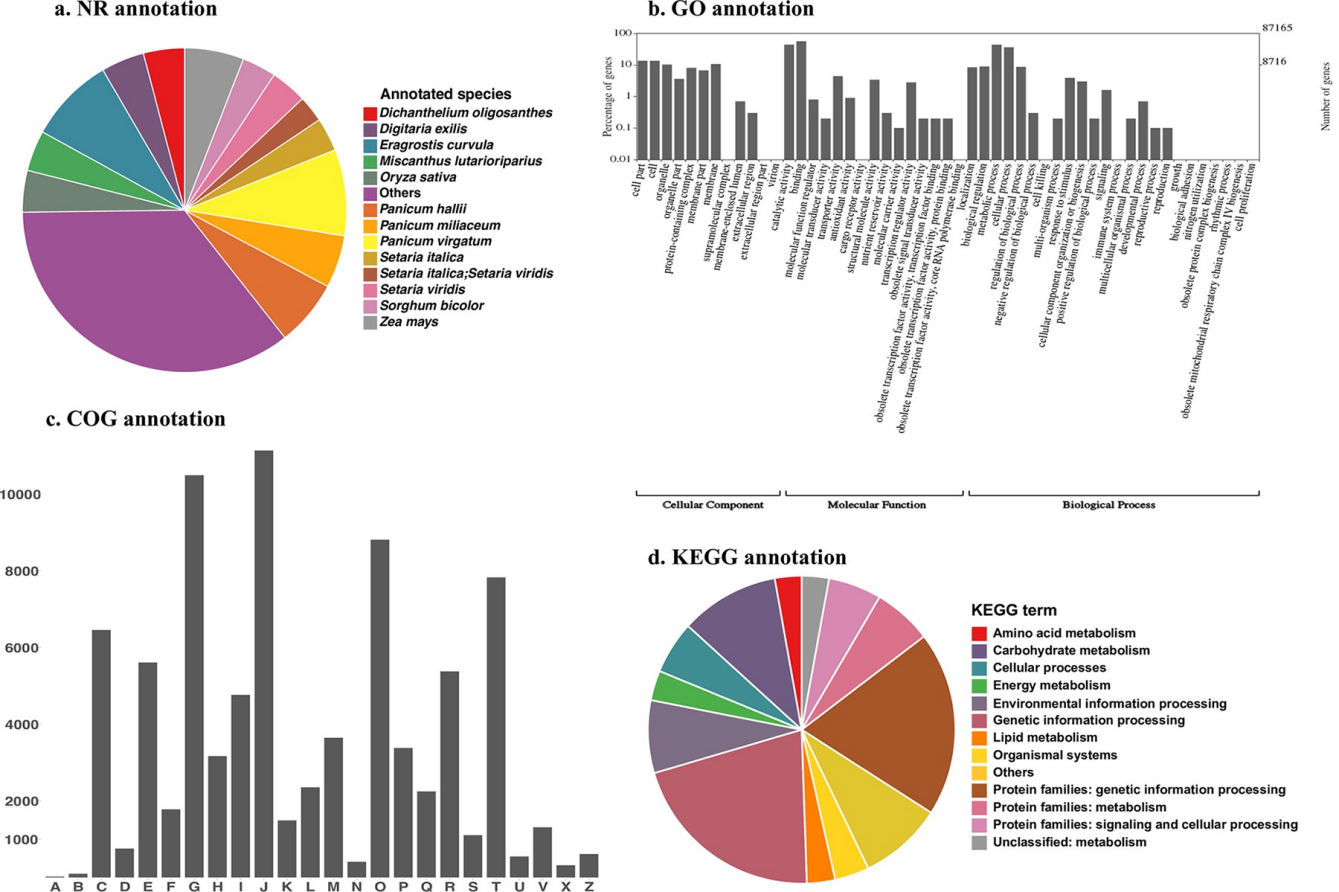
Transcriptome annotation from four database. a. Species distribution of the blastx hits for all homologous sequences against the NR database; b. Distribution of Gene Ontology (GO) classification. 87,165 transcripts have the GO annotation and are classified into 3 functional categories: cellular component, molecular function, and biological process. WEGO 2.0 (52) was used to classify GO terms and present them in this histogram figure; C. Distribution of COG functional classification by using transcriptome assembly file. 87,165 transcripts have COG annotation by using the transcriptome assembly file. The 25 categories are: [A] RNA processing and modification, [B] Chromatin structure and dynamics, [C] Energy production and conversion, [D] Cell cycle control, cell division, chromosome partitioning, [E] Amino acid transport and metabolism, [F] Nucleotide transport and metabolism, [G] Carbohydrate transport and metabolism, [H] Coenzyme transport and metabolism, [I] Lipid transport and metabolism, [J] Transition, ribosomal structure and biogenesis, [K] Transcription, [L] Replication, recombination and repair, [M] Cell wall/membrane/envelope biogenesis, [N] Cell motility, [O] Posttranslational modification, protein turnover, chaperones, [P] Inorganic ion transport and metabolism, [Q] Secondary metabolites biosynthesis, transport and catabolism, [R] General function prediction only, [S] Function unknown, [T] Signal transduction mechanisms, [U] Intracellular trafficking, secretion, and vesicular transport, [V] Defense mechanisms, [W] Extracellular structure, [Y] Nuclear structure, and [Z] Cytoskeleton; d. Distribution of KEGG terms. 95,137 transcripts have KEGG terms and are grouped based on KEGG classification. The result is from GhostKOALA [47] by using the predicted protein coding file.

To present *P*. *australis* transcriptome functional annotation, we first checked the species distribution of transcript best hit result against the NR database (Fig 3A). All top 10 species that have the most abundant matched transcripts are from the family *Poaceae* to which *P*. *australis* also belongs. Besides these species, we found some fungal species such as *Alternaria alternata*, *Alternaria tenuissima*, and *Parastagonospora nodorum* (S3 Fig). It is not an odd finding as these fungi species are commonly found in plants.

We then classified the transcripts based on their annotated GO terms (Fig 3B). In the Cellular Component category (19,486, 22.4% of all transcripts with GO terms annotation), three top GO terms have a similar number of transcripts as ‘cell part’ (11,762, 13.5%), ‘cell’ (11,762, 13.5%) and ‘organelle’ (8,973, 10.3%). There are 75,980 (87.2%) transcripts identified within the Molecular Function category. Among them, ‘binding’ (48,726, 55.9%) and ‘catalytic activity’ (38,274, 43.9%) were the most abundant. 49,355 (56.6%) transcripts are in the Biological Process category, from which ‘metabolic process’ (38,168, 43.8%) and ‘cellular process’ (31,715, 36.4%) have the largest number of matched transcripts.

Based on the annotation from the COG database, we sorted transcripts with COG match into 25 functional categories (Fig 3C). Among these categories, group G “Carbohydrate transport and metabolism” (10,487, 12.6% of all transcripts with COG annotation) and group J “Translation, ribosomal structure and biogenesis” (11133, 13.3%) are the top two categories.

In the end, we examined the annotation from the KEGG database (Fig 3D). “Genetic Information processing” from the KEGG pathway (19,761, 21.0% of all transcripts with KEGG annotation) and “Protein families: genetic information processing” from the KEGG BRITE (18,286, 19.4%) have the greatest number of transcripts in the group. With these comprehensive annotations, downstream analyses can be facilitated.

### DETs identification and GO enrichment analyses

**Fig 4:**
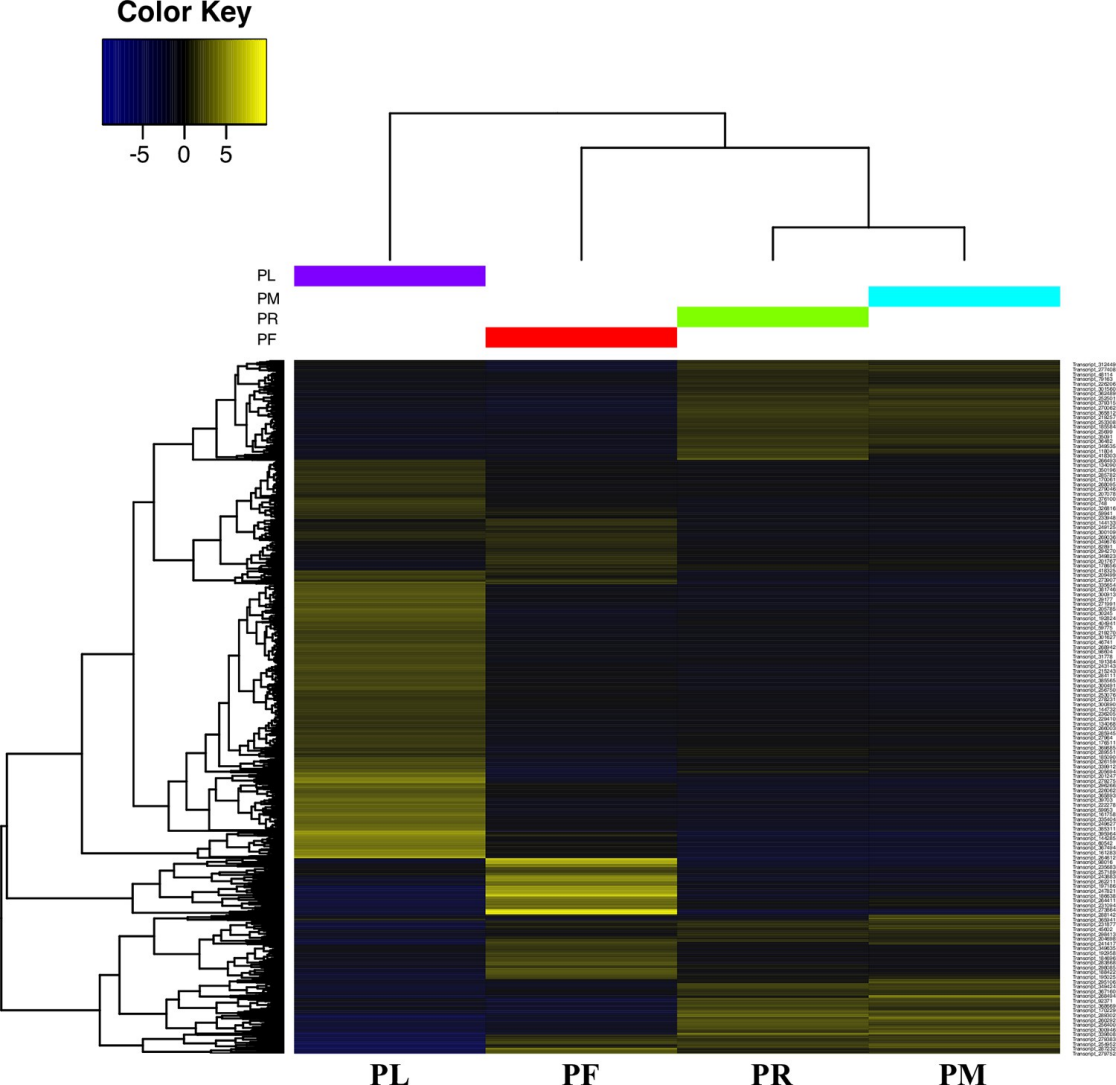
Heatmap of the expressed transcripts in all four tissues. All the transcripts with expression in all four tissues are included. Yellow depicts higher value and blue depicts lower value. PL: leaf, PF: inflorescence, PM: shoot meristem, and PR: rhizome. The set of scripts from Trinity-2.8.4 was used to generate the diagram [22].

Clean reads from each sample were separately aligned to the transcriptome assembly “cd_hit” and counted. After filtration and normalization, expressions of each transcript in all four tissues were shown in a normalized expression matrix for four tests: expression pattern for all transcripts having read counts in all four tissues (Fig 4), showing sample correlation via the heatmap (S4A Fig) and principal component analysis (PCA) plot (S4B Fig), and extracting main transcript clusters with similar expression pattern among samples (S5 Fig). They all show that transcripts in PM have a similar expression pattern as transcripts in PR. On the other hand, transcripts in PL have the most distinct expression pattern compared with the other three tissues.

**Fig 5:**
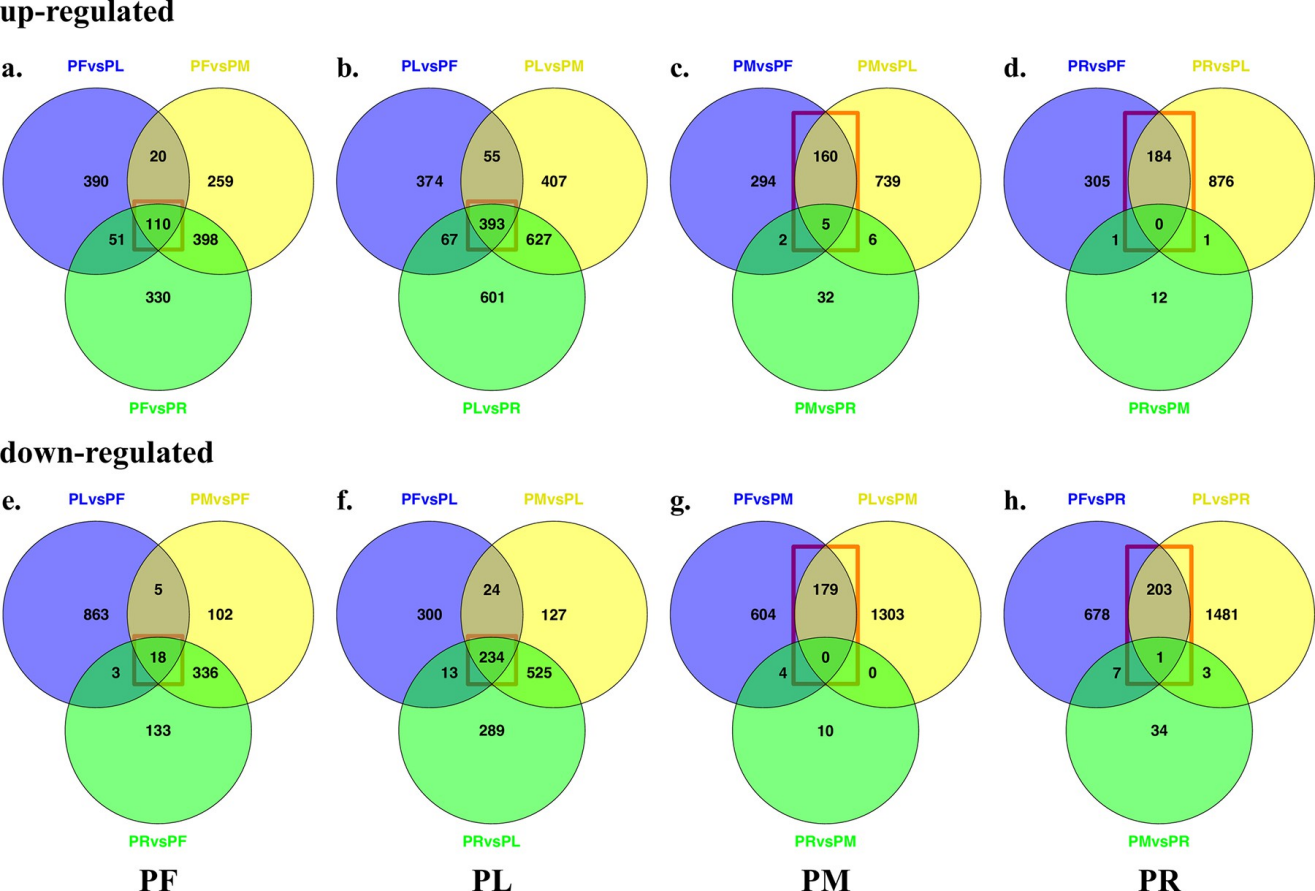
Venn gram analyses of DETs identification in each pairwise comparison among tissues. The top four diagrams (a, b, c, d) are the number of up-regulated transcripts in certain tissue from three pairwise comparisons; the bottom four diagrams (e, f, g, h) are the number of down-regulated transcripts in certain tissue from three pairwise comparisons. a and e are DETs in inflorescence (PF), b and f are DETs in leaf (PL), c and g are DETs in shoot meristem (PM), d and h are DETs in rhizome (PR). Red boxes are the number of tissue-specific DETs for the following analyses.

Analyses to identify the DETs in each pairwise comparison among tissues were conducted. The number of identified DETs in each comparison is counted, compared, and shown in the Venn diagrams (Fig 5 and S1 Data). As two examples, we use DETs identified in PL and PR. In PL, there are 1406 DETs between PL and PF (889 up-regulated and 571 down-regulated in PL), 2392 DETs between PL and PM (1482 up-regulated and 910 down-regulated in PL), and 2741 DETs between PL and PR (1688 up-regulated and 1061 down-regulated in PL). In PR, 1,379 DETs are found in the comparison between PR and PF (490 up-regulated and 889 down-regulated in PR), and 59 DETs are identified in comparison between PR and PM (14 up-regulated and 45 down-regulated in PR). We then performed enrichment analyses of GO terms for these DETs identified in each pairwise comparison (S6 Fig and detailed information of GO terms with false discovery rate (FDR) < 0.05 in S2 Data). Results from up-regulated DETs found in the above two examples are demonstrated here: 1. From top GO terms linked to PL up-regulated transcripts in all three pairwise comparisons (PL vs. PF, PL vs. PM, and PL vs. PR), we found GO:0009772 (photosynthetic electron transport in photosystem II, FDR successively as 2.61*10^−14^, 1.08*10^−13^ and 4.81*10^−11^) and GO:0009521 (photosystem CC, FDR successively as 2.23*10^−13^, 2.20*10^−15^ and 9.68*10^−15^); 2. As transcripts have similar expression patterns in PR and PM, only 14 transcripts up-regulated in PR based on the PR vs. PM comparison. We could only find GO terms with significant FDR in the other two pairwise comparisons (PR vs. PL and PR vs. PF), in which linked transcripts are up-regulated in PR. GO:1901566 (organonitrogen compound biosynthetic process, FDR = 9.68*10^−6^ and 2.34*10^−13^) and GO:1901576 (organic substance biosynthetic process, FDR = 6.89*10^−6^ and 9.90*10^−13^) are on the top of GO terms list. These results are consistent with expected biological processes and organ function, given that leaves act as principal photosynthetic structures and roots/rhizomes act as nutritive storage organs and propagules for regeneration.

To determine tissue-specific DETs, we extracted DETs that were found in all three pairwise comparisons involved with each tissue (S3 Data). For example, tissue-specific DETs in PL are the ones detected in all three pairwise comparisons (PL vs. PF, PL vs. PM, and PL vs. PR). Because transcripts show similar expression patterns in PR and PM, we chose the tissue-specific DETs in these two tissues from the overlap between two pairwise comparisons only with PF and PL but not PR or PM. Overall, there are 128 specific PF DETs (110 up-regulated and 18 down-regulated), 627 specific PL DETs (393 up-regulated and 234 down-regulated), 342 specific PM DETs (165 up-regulated and 179 down-regulated), and 398 specific PR DETs (184 up-regulated and 204 down-regulated) (Red box shown in Fig 5). The detailed information of tissue-specific DETs can be found in S3 Data. Similarly, we separately performed enrichment analyses of GO terms on these different types of tissue-specific DETs. Except for the analysis on down-regulated transcripts in PL, for which we could not find any GO terms with significant FDR, the other seven GO terms enrichment analyses linked to the certain type of tissue-specific DETs are shown in S6 Fig and detailed information with FDR < 0.05 is on S4 Data. Even though the background is not the same as previous GO terms enrichment analyses on transcripts from each pairwise comparison among tissues, the results are similar: GO:0009772 (FDR = 7.16*10^−16^) and GO:0009521 (FDR = 5.82*10^−11^) are also enriched for PF specific up-regulated transcripts; GO:1901566 (FDR = 2.03*10^−4^) and GO:1901576 (FDR = 4.37*10^−5^) are found in GO terms enrichment for PR specific up-regulated transcripts as well.

### Putative TFs identification

**Fig 6:**
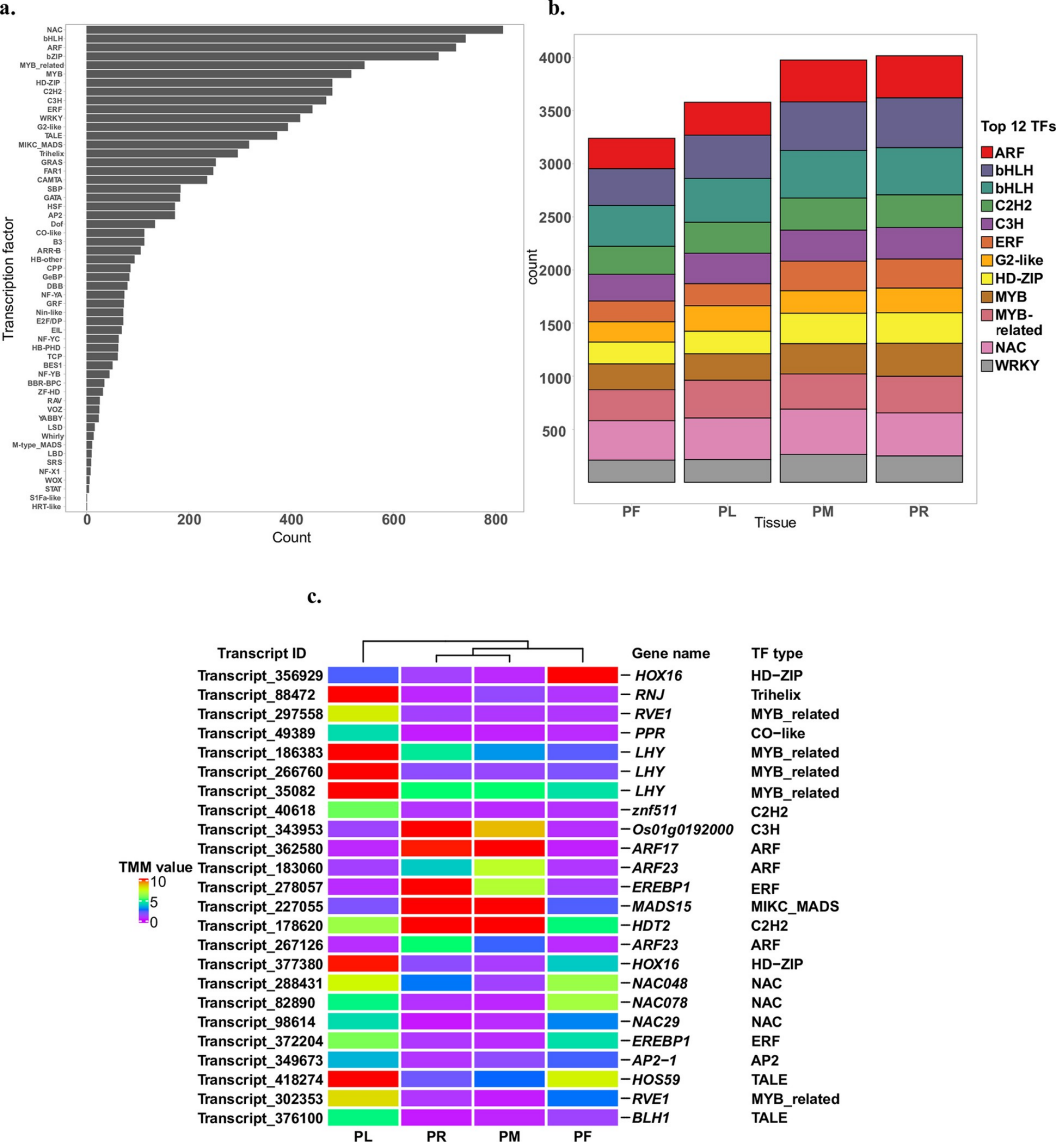
TFs identified in *P*. *australis* transcriptome assembly “cd_hit”. a. The distribution of all 55 types of putative TFs in *P*. *australis* transcriptome; b. The number of the top 12 types of putative TFs in each tested tissue. If the TF has the expression in that tissue from the normalized expression matrix, it would be counted; c. Heatmap of the TFs showing the tissue-specific expression pattern. Putative types of TFs and matched gene names are listed as well, which are from the UniProt-Swissprot annotation. Heatmap was generated from the R package ComplexHeatmap [53]. Red depicts a higher TMM value and purple depicts a lower TMM value. PL: leaf, PF: inflorescence, PM: shoot meristem, and PR: rhizome.

TFs are essential regulatory elements for gene expression, which can be responsible for tissue-specific gene expression patterns and tissue-specific development. TFs identified in *P*. *australis* transcriptome might give us a hint to reveal why non-native subspecies are invasive. In the end, a total of 10,828 putative TFs are identified in the transcriptome. Among 55 types of TFs identified, the top 12 types of TFs that account for the majority of the group (62.0% of the whole putative TFs) are NAC (815), bHLH(742), ARF(723), bZIP (689), MYB_related (544), MYB (518), C2H2 (481), HD-ZIP (481), C3H (469), ERF (442), WRKY (418), G2-like (394) (Fig 6A and S5 Data). We then examined the number distribution of these top 12 groups in each tissue, depending on whether the putative TF has the TMM (Trimmed Mean of M) value on that tissue from the normalized expression matrix obtained from the previous analysis. By comparison, both the total number of all 12 types and the number of each type of TFs vary among four tissues (Fig 6B). Interestingly, the rank of PF is not only the lowest in the comparison of the total number of 12 types of TFs but also the lowest in each type. For these types of TFs, we further separately performed the goodness-of-fit test to check whether their distributions among tissues are different. After calculation, the total number and six individual types of TFs (bHLH, ARF, MYB, WRKY, HD-ZIP, and ERF) show statistical significance (P < 0.05, S2 Table in S1 File). This result may demonstrate that tissues have their specific TFs to regulate gene expression for development.

Twenty-four putative TFs are found to have differentially expressed patterns among four tissues by comparing the list of putative TFs with the list of tissue-specific DETs (Fig 6C). Only one putative HD-ZIP (transcript_356929) has a significantly higher expression in PF. Seven putative TFs are up-regulated in PL. The remaining putative TFs are differentially expressed in PM and PR. Some putative TFs are simultaneously on the list of up-regulated TFs in one tissue and the list of down-regulated TFs in the other tissue. These putative TFs with tissue-specific expression patterns may have important roles in that certain tissue. For example, transcript_343953 is down-regulated in leaf and inflorescence tissue. In rice, its annotated gene *Os01g0192000* is also down-regulated during natural leaf senescence, panicle development, and pollination [53]. Therefore, based on the possible role of *Os01g0192000* as a negative regulator in leaf senescence of rice, it is plausible to suggest a similar role of transcript_343953 in *P*. *australis*.

### Candidate SSRs identification

**Fig 7:**
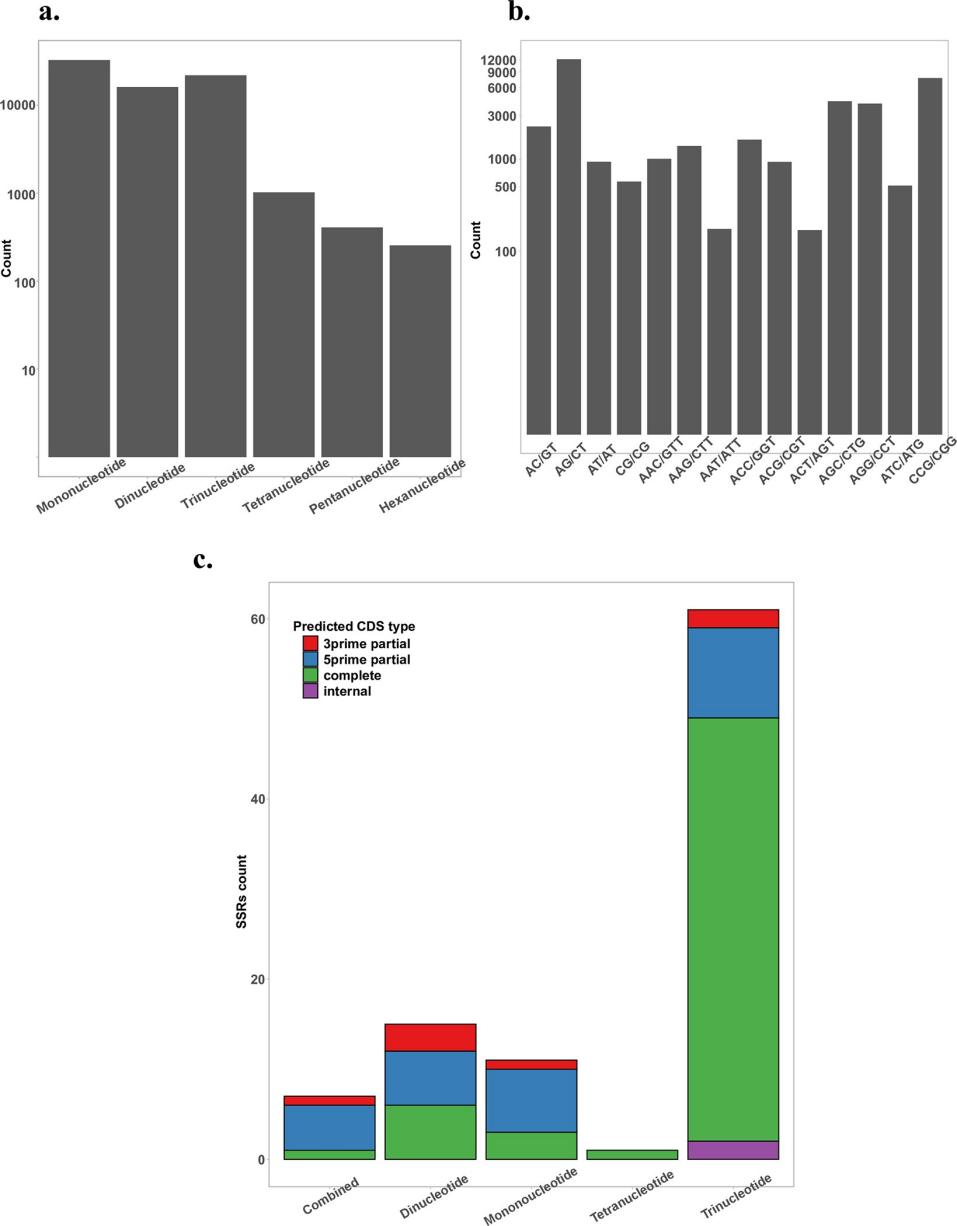
SSRs identified in *P*. *australis* transcriptome assembly “cd_hit”. a. Distribution of all SSRs categories in *P*. *australis* transcriptome; b. Distribution of all subcategories of dinucleotide and trinucleotide in *P*. *australis* transcriptome; c. Distribution of CDS-SSRs in *P*. *australis* transcriptome. It is based on the number of each category of SSRs, and each category is stacked by the type of candidate coding region. Combined is the SSRs with more than one repeats in the closed location of the sequence.

Microsatellites, commonly referred to as SSRs, are still commonly and effectively used as a genetic marker system in the study of plant population genetics. In the recent work on *P*. *australis*, eight SSRs markers from plastid DNA were used to characterize the genetic diversity of native and non-native *P*. *australis* subspecies in Wisconsin, US [54]. Meanwhile, using *de novo* assembled transcriptome to identify SSRs has been tried in many plant species such as *Lilium* species, *Menispermum* species, *Populus wulianensis*, *Fraxinus velutina*, and *Idesia polycarpa* [55–59]. Most of the studies also proved the validity of these predicted SSRs markers by PCR. Therefore, via using transcriptome assembly “cd_hit” and the MISA microsatellite finder [52], additional candidate SSRs markers can be detected for future genetic diversity studies on *P*. *australis*. Within the transcriptome assembly, a total of 72,165 candidate SSRs were identified in 59,516 transcript sequences. Among these candidate SSRs, mononucleotide is the largest fraction (32,588, 45.2%), followed by trinucleotide (21,841, 30.3%), dinucleotide (16,051, 22.2%), 1,022 tetranucleotide (1,022), pentanucleotide (408), and hexanucleotide (255) (Fig 7A). We further viewed the distribution of subcategories among dinucleotide and trinucleotide repeats based on their nucleotide composition (Fig 7B). AG/CT represents the largest fraction of dinucleotide repeats (12,271, 76.5%), which is consistent with the findings of other plant studies using similar methods [55, 56, 58, 60]. On the other hand, the distribution of trinucleotide repeats identified in *P*. *australis* is like the results in the *Phragmites karka* study [3]. CCG/CGG is the most abundant of the group (7,671, 35.1%), which is followed by AGC/CTG (4,279, 19.6%) and AGG/CCT (4,036, 18.5%).

We further compared the list of transcripts having candidate SSRs with the list of tissue-specific DETs. There are 356 transcripts with candidate SSRs markers showing tissue-specific expression patterns (S6 Data). We then checked these 356 transcripts with the coding region file generated by TransDecoder to select the candidate transcripts, within which SSRs are in the predicted protein coding region. In the end, 95 transcripts having candidate CDS-SSRs are selected. The annotated gene and protein names of these 95 candidate CDS-SSRs are marked in S6 Data as well. The major category of candidate CDS-SSRs is trinucleotide (61, 64.2%, Fig 7C). Among them, the most abundant subcategory is still CCG/CGG (31, 50% of the trinucleotide category, 32.6% of the candidate CDS-SSRs). This result supports the observation that the predominance of SSRs in coding regions is trinucleotide category [61, 62], likely due to the selection pressure against mutations that alter the reading frame. In addition, it is known that the polymorphic SSRs located in the CDS region could modify the corresponding protein, and SSRs located in UTRs or introns could affect the relative expression of the gene [55, 61]. With the help of transcriptome functional annotation, we may link the candidate CDS-SSRs to certain traits of the non-native *P*. *australis* subspecies since transcripts with these CDS-SSRs also show tissue-specific expression patterns.

### Predicted herbicide and salinity resistant transcripts analyses

**Fig 8:**
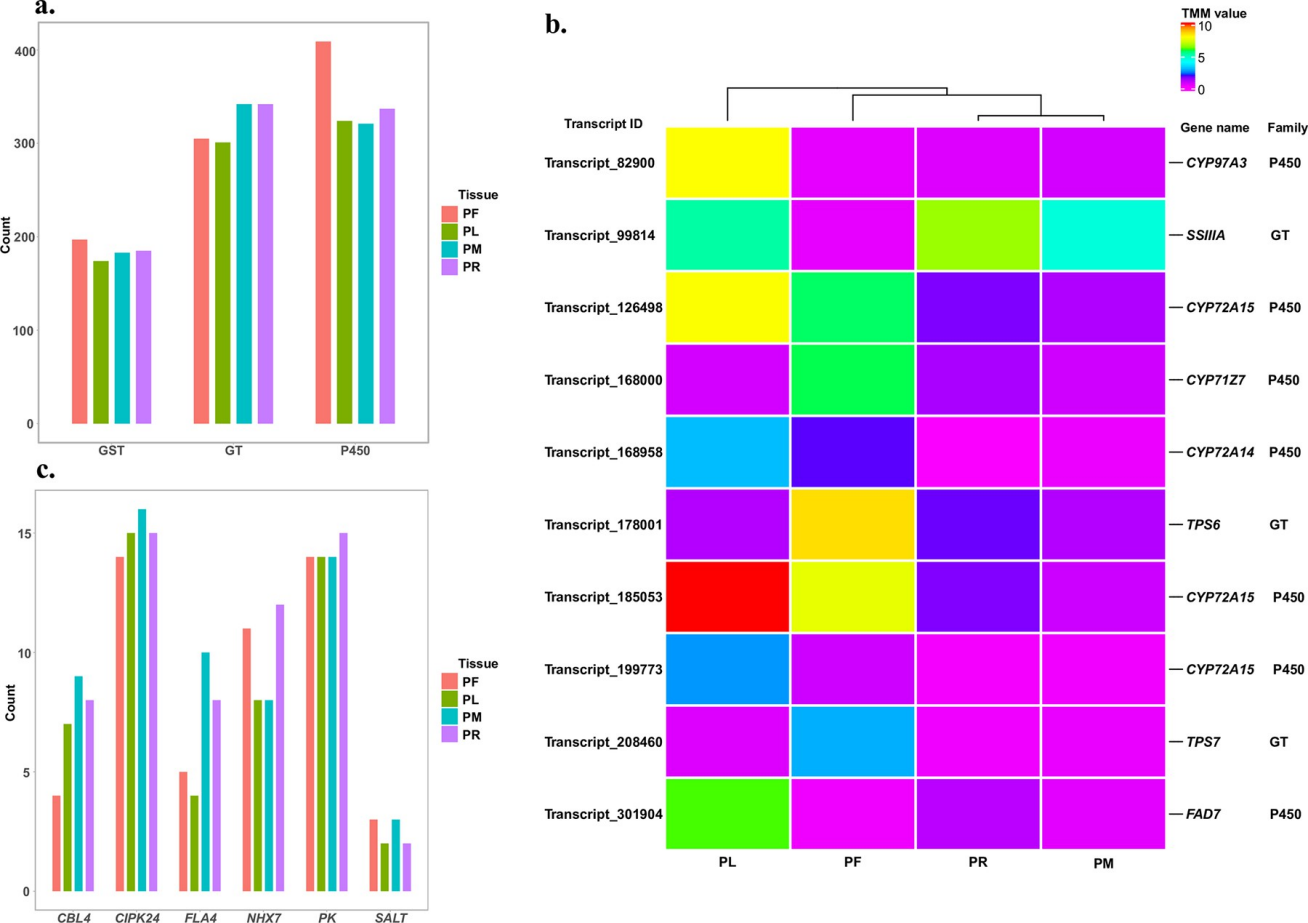
Identification of herbicide- and salinity-resistant genes and analyses. a. Distribution of transcripts with the domain from three types of the herbicide-resistant gene families among four tissues in *P*. *australis* transcriptome; b. Distribution of transcripts annotated as six types of the salinity-resistant genes among four tissues in *P*. *australis* transcriptome; a-b, if the transcript has the expression in that tissue from the normalized expression matrix, it would be counted; c. Heatmap of the transcripts predicted as herbicide-resistant genes with tissue-specific expression patterns. Gene name is from the UniProt-Swissprot annotation. Heatmap was generated from the R package ComplexHeatmap [53]. Red depicts a higher TMM value and purple depicts a lower TMM value. PL: leaf, PF: inflorescence, PM: shoot meristem, and PR: rhizome.

Herbicide is a common management treatment for *P*. *australis* [6, 7] and there is a growing concern about increased resistance through time, but we don’t know very much about the genes involved in herbicide-resistance mechanisms. Three herbicide-resistant gene families which include cytochrome P450, GT, and GST has been described in *P*. *australis* [63]. In the study of *de novo* transcriptome of *Apera spica-venti*, these three gene families are also abundant in all tested tissues with the potential to resist herbicide [64]. In this study, as a demonstration to use the transcriptome and its annotation, we screened candidate transcripts with domains of these three gene families. After analysis, all of them have multiple matched transcripts (649 cytochrome P450s, 368 GSTs, and 557 GTs), but the distribution patterns of these three herbicide-resistant gene families are not similar based on the normalized expression matrix. Though cytochrome P450s are found in the greatest number within the transcriptome, it is not always the case in individual tissue. Unlike the other two families that have the most matches in PF, the tissues that have the most matches of GTs are PM and PR (both are 342). This number is even bigger than the number of cytochrome P450 found in PM and PR (321 and 337) (Fig 8A). Detailed information of the identified transcripts related to herbicide-resistant could be found in S7 Data. From these 1,574 matched transcripts, we further found 10 transcripts showing tissue-specific expression patterns. Compared with other identified transcripts, only Transcript_99814 has a relatively higher expression in PR and PM, and it is predicted to have a GT domain (Fig 8B). Based on the distribution result and the tissue-specific expression pattern result, it is suggested that GTs might play a more important role in herbicide resistance of *P*. *australis* rhizome and shoot meristem.

Salinity tolerance is another large topic of interest in *P*. *australis* research [13, 14]. For non-native *P*. *australis* subspecies, salinity tolerance is important for plant invasiveness [65]. Therefore, as another application, we could detect several genes associated with the salt stress response. The genes used in this search include *NHX7*, *CIPK24*, *CBL4*, *PK*, *FLA4*, and *SALT*, which are based on the list summarized by GD Holmes et al [14] (S3 Table in S1 File). Applying similar analyses in the herbicide-resistant study, we found 12 transcripts annotated as *NHX7*, 22 as *CIPK24*, 9 as *CNBL4*, 19 as *PK*, 16 as *FLA4*, and 3 as *SALT* (S8 Data). After comparing the distribution of these salinity-related transcripts among tissues, more transcripts matched with these six genes are expressed in below-ground tissue (PR and/or PM) in comparison with the aboveground tissues (Fig 8C). Even though there is no transcript showing tissue-specific expression pattern, it may still demonstrate their tissue-specific roles in adaptations to a saline environment.

## Discussion

Many bioinformatics software tools and methods have been designed with the development of the RNA-seq as an experimental approach. In *de novo* transcriptome assembly, the choice at each step can affect the quality of the transcriptome and downstream analyses. Even the selection of the programs to trim adaptor sequences from raw reads requires careful consideration [17]. More complex assembly tools using different algorithms might cause a similar problem. Trans-AbySS [28], SOAPdenovoTrans [27], and Trinity [23] are de Bruijn graph-based; the algorithm of Shannon [26] is based on information theory. The expectation that different algorithm-based tools would produce different results is born out in the results from our transcriptome assemblies’ quality assessments (Fig 3) and other studies [16, 18]: with the same setting and treatment, transcriptome assemblies generated by different assembly tools still show distinct characteristics. Kmer selection is another important factor for consideration in *de novo* transcriptome assembly. In theory, “the shorter kmer setting would utilize most of the reads and a relatively completed assembly would be built on these. However, a higher proportion of misassemblies, joins, retained introns, indels, and frameshifts would also be generated. The longer kmer setting would use fewer reads but tend to have fewer mistakes than short kmer assemblies” [30]. In reality, differences do exist among transcriptome assemblies analyzed by different kmer settings [18, 19]. To solve this problem, we combined transcriptome assemblies constructed by the same tool but with different kmer settings as one file and refined it via CD-HIT-EST. Studies that collated multiple transcriptome assemblies from different tools as one non-redundant transcriptome assembly via the EvidentialGene tools found that a transcriptome assembly with reliably accurate gene sets could be generated [16–20]. We applied the same method but generated two non-redundant transcriptome assemblies in two separate ways: with or without treatment by CD-HID-EST. From the above statement and our observation that differences would be easily generated during *de novo* transcriptome assembly, it is necessary to construct multiple transcriptome assemblies via different methods, perform quality assessments, and find the optimal transcriptome assembly for a certain purpose. In this study, our purpose was to construct the transcriptome assembly with more biologically accurate and complete transcripts that would facilitate the functional annotation of transcripts and downstream analyses. Therefore, transcriptome assembly “cd_hit” might not be the best transcriptome assembly in other aspects, as it may neglect non-coding RNAs. Yet, it is the optimal transcriptome assembly among forty-nine transcriptome assemblies we constructed for this study. All downstream analyses also prove the validity of this transcriptome assembly. One thing we noticed during the transcriptome assembly is that all the seven compared transcriptome assemblies have many transcripts and a high number of duplicated copies as found in two BUSCOs assessments, except for transcriptome assemblies “Trinity_GG”. This characteristic may be due to the level of polyploidy in the genome of *P*. *australis* or an artifact of how we collected samples directly from an open field. Several studies reported that the invasive non-native subspecies is known from the European populations and belongs to the tetraploid lineages [2, 4].

To improve the annotation of this optimal transcriptome assembly, we included the results from the annotated protein file of *S*. *italica* v2.2 in addition to other commonly used databases. *P*. *australis* belongs to Arundinoideae subfamily, which is the sister subfamily of Panicoideae where *S*. *italica* is grouped. A recent work confirmed the closed relationship between *P*. *australis* and *S*. *italica* based on multiple amino acid alignments [66]. Since *S*. *italica* is well annotated [24], we could delve into the putative function of interesting transcripts more deeply. The CSV file including all thirteen types of annotations can be downloaded from the webpage and easily viewed via Microsoft Excel. This information will be a valuable resource for future *P*. *australis* studies. The identification of predicted herbicide- and salinity-resistant transcripts exemplifies the application of this resource. Compared with other predicted transcripts from herbicide-resistant analysis, we find that transcript_99814 with a GT domain is the only one showing a relatively higher expression in PM and PR. The annotated gene of this transcript is *SSIIIA* from *Oryza sativa subsp*. *japonica*, which is involved in starch synthesis. It is known that GTs could respond to a variety of plant stresses by conjugating with various phytohormones and other metabolites through attachment to the activated sugars [67]. In a study of *P*. *australis*, Kanai et al. [68] found that starch content increases most in the shoot and binds to Na+ in response to salt stress. The previous evidence and our findings indicate the possible function of GTs in *P*. *australis* under stress. The higher expression of transcript_99814 may also reflect the metabolism of the rhizome and shoot meristem as the energy storage organs, as these two functions may not be exclusive. Previous studies on *P*. *australis* also point out the role of the other two gene families P450s and GSTs in the detoxification of herbicide in addition to GTs [63]. Although we could not find any transcripts related to these two families having tissue-specific expression patterns in PM and PR, these kinds of transcripts do show significantly higher expression in PL or PF. Both leaf and inflorescence tissues would be the most exposed tissues to aerial herbicide treatment. Further studies are needed to test the role of these three superfamilies in the detoxification of herbicide on the molecular level among different types of tissues. Alternatively, all predicted salinity-resistant transcripts identified in our analysis did not show tissue-specific expression patterns. This finding is consistent with the previous study on leaf samples, in which the same type of salinity-resistant genes also did not show significant expression changes under different salinity treatments [14]. However, as all the six types of salinity-resistant genes have more matched transcripts expressed in PR and/or PM, these differences among tissues might still explain the function of these salinity-resistant transcripts in *P*. *australis*.

RNA-seq studies could also be used to reveal the epiphytic and endophytic communities within the plant ecology without applying a culturing method [69, 70]. Research has reported that the symbiotic relationship between Epichloë fungal endophytes in cool-season grasses may improve their abiotic and biotic resistance [71]. Researchers also have tried to use microbiomes to control invasive non-native *P*. *australis* invasive [72]. In this study, we identified transcripts annotated as genes from some fungal and insect species, even though the abundance of such matched transcripts is not great (S3 Fig). Genus *Alternaria* (*A*. *alternata* and *A*. *tenussima*) has the most non-plant matched transcripts, which is consistent with studies on *Phragmites* using the culture method [73, 74]. *Stagonospora sp*. endophyte has been reported to increase *Phragmites* seedling biomass production in the European population [75]. We find 2,609 matched transcripts of this species. Unlike reports in previous studies using the culture method, we identified 4,163 transcripts annotated as *Parastagonospora nodorum SN15*. This species is the fungal pathogen of wheat and the causal agent of *Septoria nodorum* blotch. We also found that transcript_409068 belonging to this fungus group is down-regulated in the leaf by comparing the list of tissue-specific DETs. The Uniprot-Swissprot annotation shows that it is likely FAD-biding monooxygenase *ktnD*, which mediates the biosynthesis of the bicoumarin kotanin. A previous study demonstrated that Orlandin, a member of the class kotanin, could inhibit plant growth [76]. It is implied that non-native *P*. *australis* subspecies might have an immune strategy to the infection of this pathogenic fungus. Studies on this aspect may provide insights into the success of invasive plant species and a future research direction for *Phragmites* management. Using these above non-plant transcripts groups and transcripts annotated as from another insect group *Contarinia nastrutii*, we separately checked their distribution among tissues. PF has the most abundant matched transcripts expressed for all these five species (S3 Fig). This distribution pattern reflects the colonization of these tissues and structures within the context of the ecological communities. The comparatively soft tissue versus the other three tissues and its high, exposed position in the environment make the inflorescence a primary target for fungal infection and insect herbivory.

Due to the lack of tissue replicates, identified DETs may not accurately display the expression differences of transcripts among tissues; however, findings from tissue-specific DETs and the followed GO terms enrichment analyses of these two types of DETs do demonstrate the utility of the results. In addition to GO:1901566 and GO:1901576, which we previously discussed, other GO terms, such as GO:0009058 (biosynthetic process) and GO:0044249 (cellular biosynthetic process) are also in the enrichment list of PR and PM from both GO enrichment analyses (S2 and S4 Datas). These GO terms all refer to different kinds of biosynthetic processes that reflect the function of specific organ. Though PM and PR share the same types of GO terms, the transcripts linked to the GO terms that are enriched in PM are not always the same as those found in PR. The common transcripts might reflect the similar connection between rhizome and shoot meristem. For example, some of the common transcripts are annotated as ribosomal proteins such as 40S ribosomal protein S7, 40S ribosomal protein S11, and 60S ribosomal protein L13-2. In rice, ribosomal protein genes were found to show differential expression under abiotic and biotic stresses [77]. On the other hand, we find that some transcripts are only in one tissue list. For example, transcript_349990 only appears on the GO enrichment list of PR and is predicted as bifunctional dihydrofolate reductase-thymidylate synthase. A previous study found that nitric oxide synthase uses tetrahydrofolate as a cofactor to respond to abiotic stress in *Arabidopsis* [78]. The match of this transcript in *S*. *italica* is *Seita*.*2G410800*, which also shows a higher expression in the root than the shoot of the plant from the Phyzome v13 database [79]. The dissimilarity between rhizome and shoot meristem might be revealed in future studies focusing on this group transcripts.

The distributions of putative TFs are not similar among our study and other studies on *Phragmites* [1, 3, 15]. Several factors could explain this dissimilarity: 1. The tissues used for transcriptome assembly are not the same. As we observed that the number of all types of putative TFs varies among tissues, using different types of tissues would alter the total number of TFs identified in the transcriptome assembly (Fig 6B). This variation in transcript identification highlights the importance of using multiple types of tissues to provide the transcriptomic information; 2. The quality of the assembled transcriptome would affect the results of TFs identification as well. As we have shown, the utilization of multiple assembly tools and settings can greatly affect the quality of the generated transcriptome; 3. The cut-off e-value and selection of species database used in BLAST searches differed. Yet, despite these possible causes, TFs found in the top groups are still similar in all the studies, including bHLH, bZIP, C3H, MYB_related, and NAC. Most of these types of TFs are also the most abundant TFs in *S*. *italica* [51]. We identified seven putative TFs having significantly higher expression in PR and PM. Though they are not the main types of TFs showing differential expression in the leaf under salinity treatment [13], their predicted function might reflect tissue-specific development rather than directly displaying the difference in the specific tissue in response to the environment. In addition to transcript_343953, we identified two additional transcripts that may reflect tissue-specific development: transcript_362580 (annotated gene *ARF17*) and transcript_227055 (annotated gene *MADS15*). Based on previous studies, *ARF17* has a potential role in the regulation of adventitious root development in *Arabidopsis* [80]; Similarly, in rice, *MADS15* regulates the expression of genes related to some key transporter traits (SWEET3A, MDR-like ABC, and vacuolar iron transporter homolog 2) and the expression of genes related to the root traits [81].

The distribution of SSRs categories of *P*. *karka* generalized by SS Nayak et al. [3] differs from our SSRs distribution in *P*. *australis*, although both studies found more trinucleotides repeats than dinucleotide repeats. In their findings, tetranucleotide repeats are also more abundant than dinucleotide repeats. This difference may be caused by several of the following factors: different species, less stringent parameters for tetranucleotide used in MISA for *P*. *karka* work (5 vs 3 repeats for tetranucleotide), and distinct strategies to assemble the transcriptome. Sonah et al [62] used the same tool and parameters as we did to check the distribution of SSRs in three *Poaceae* plants (*Brachypodium*, sorghum, and rice) but via using the genome information. Their results are like ours in *P*. *australis*: mononucleotide, dinucleotide, and trinucleotide repeats are always the major three categories. Similarly, their results about the most abundant subcategory of each SSRs category are also the same as those we found (AG/CT in dinucleotide and CCG/CGG in trinucleotide). For CDS-SSRs, we noticed that nucleotide numbers of 14 non-trinucleotide members are multiples of three, which means they also could be considered as potential in-frame indels. In other words, there could be a total of 75 CDS-SSRs as in-frame indels (78.9%), although they may be still underrepresented under the limitation of the transcriptome. In three *Poaceae* plant genomes, trinucleotide repeats contributed to about 93% of CDS-SSR [62]. Meanwhile, we found that there are more transcripts with CDS-SSRs showing up-regulated expression in PL than transcripts showing other tissue-specific expression patterns. Two reasons may explain it: 1. Initially, PL has more up-regulated transcripts (Fig 5); 2. transcripts annotated as organelle genes are normally up-regulated in PL. Unlike other tissues, it would provide an extra pool to select transcripts with SSRs, since we find more than half of the transcripts in the PL CDS-SSRs list are predicted as from chloroplast (S6 Data).

## Conclusion

Though *P*. *australis* is among the most studied invasive plant species [9], future studies are still necessary to describe characteristics such as intraspecific hybridization between native and non-native subspecies, genome size and ploidy level related to plant traits, microbial association diversity between *P*. *australis* subspecies, and ecological aspects of devastating and beneficial consequences by *Phragmites* invasion. The use of *Phragmites* for novel purposes including phytoremediation of pharmaceuticals, engineering abiotic stress-tolerant plants, and bioenergy presents additional challenges. In this study, we demonstrated that transcriptome construction can impact the quality and value of the genomic product. Even though short length RNA-seq data without any biological or technical replicates could impose a limitation on the *de novo* transcriptome assembly and downstream analyses, our work to select the optimal transcriptome and perform a comprehensive functional annotation, similar to the two applications we presented, can still provide a valuable resource to help works to address the genetic mechanisms underlying the aggressive and successful invasiveness of *P*. *australis*.

## Supporting information

S1 File (DOCX)

S1 Fig Length distribution of transcripts from seven constructed transcriptomes generated by rnaQUAST. (PDF)

S2 Fig Length distribution of candidate coding regions predicted by TransDecoder. (PNG)

S3 Fig Distribution of five main groups of non-plant transcripts among four tissues. They are all from the NR blastx annotation. PL: leaf, PF: inflorescence, PM: shoot meristem, and PR: rhizome.
(PDF)

S4 Fig Sample correlation based on transcripts expression matrix. a. Heatmap shows the samples’ correlation based on the transcript’s expression matrix. Yellow depicts high value and blue depicts lower value; b. PCA plot based on the transcript’s expression matrix. PL: leaf, PF: inflorescence, PM: shoot meristem, and PR: rhizome.
(PDF)

S5 Fig Main clusters of transcripts extracted based on expression matrix. Six main clusters (a to f) having similar expression patterns in four tissues were extracted by Trinity script define_clusters_by_cutting_tree.pl. The number of transcripts in each cluster is listed after the cluster name in the title.
(PDF)

S6 Fig Scatter plot for GO enrichment analysis on DETs in each pairwise comparison among tissues. a. GO enrichment analysis on up-regulated DETs in PF between PF and PR; b. GO enrichment analysis on up-regulated DETs in PF between PF and PM; c. GO enrichment analysis on up-regulated DETs in PF between PF and PL; d. GO enrichment analysis on up-regulated DETs in PL between PL and PM; e. GO enrichment analysis on up-regulated DETs in PL between PL and PR; f. GO enrichment analysis on up-regulated DETs in PL between PL and PF; g. GO enrichment analysis on up-regulated DETs in PM between PM and PF; h. GO enrichment analysis on up-regulated DETs in PM between PM and PL; i. GO enrichment analysis on up-regulated DETs in PM between PM and PR; j. GO enrichment analysis on up-regulated DETs in PR between PR and PF; k. GO enrichment analysis on up-regulated DETs in PR between PR and PL; l. GO enrichment analysis on up-regulated DETs in PR between PR and PM. The size of the circle indicates the transcript number. Each different color indicates a different FDR value.
(PDF)

S7 Fig Scatter plot for Go enrichment analyses on tissue-specific DETs. a. GO enrichment analysis on up-regulated DETs specific in PF; b. GO enrichment analysis on down-regulated DETs specific in PF; c. GO enrichment analysis on up-regulated DETs specific in PL; d. GO enrichment analysis on up-regulated DETs specific in PM; e. GO enrichment analysis on down-regulated DETs specific in PM; f. GO enrichment analysis on up-regulated DETs specific in PR; g. GO enrichment analysis on down-regulated DETs specific in PR. The size of the circle indicates the transcript number. Each different color indicates a different FDR value.
(PDF)

S1 Data The lists of transcripts with annotation. Transcripts are generated from the pairwise comparisons among tissues.
(XLSX)

S2 Data The lists of results in each GO terms enrichment analysis on transcripts from the pairwise comparisons among tissues. (XLSX)

S3 Data The lists of tissue-specific DETs with annotation. (XLSX)

S4 Data The lists of results in each GO terms enrichment analysis on tissue-specific DETs. (XLSX)

S5 Data The list of putative TFs expressed in each tissue with annotation. (XLSX)

S6 Data The lists of transcripts having SSRs and showing tissue-specific expression patterns with annotation. The transcripts with CDS-SSRs were marked with predicted gene names and protein names.
(XLSX)

S7 Data The lists of putative herbicide-resistant transcripts with annotation. (XLSX)

S8 Data The list of putative salinity-resistant gene transcripts with annotation. (XLSX)
