# Supplementary material for: Optimization and application of non-native Phragmites australis transcriptome assemblies
Source: PLoS One. 2023 Jan 23;18(1):e0280354. doi: 10.1371/journal.pone.0280354 (PMC9870158; doi:10.1371/journal.pone.0280354)
Supplement: S1 File — (DOCX) [file pone.0280354.s001.docx]

**Supplement Material**

**S1 Table. Statistics of remaining** **transcriptome assessments for seven constructed transcriptomes**

|  | Mappi-ng  rate | Refere-nce  leaf  Mapping  rate | Refere-nce rhizome  Mapping  rate | Transcri-pts  >500bp | Transcri-pts  >1000bp | Maximum transcript  length | DETONA-TE  score |
| --- | --- | --- | --- | --- | --- | --- | --- |
| Trinity | 92.39% | 91.24% | 90.61% | 134,024 | 56,236 | 19,576 | -1.77*10^10^ |
| Trinity_GG | 53.42% | 44.51% | 41.7% | 17,108 | 4,828 | 17,078 | -2.38*10^10^ |
| GapClosed | 95.12% | 92.89% | 92.3% | 150040 | 48166 | 37,456 | -1.89*10^10^ |
| Transabyss | 93.81% | 92.85% | 92.31% | 183,897 | 60,338 | 42,430 | -1.67*10^10^ |
| Shannon | 93.11% | 91.5% | 90.85% | 326,806 | 184,621 | 64,081 | -1.67*10^10^ |
| cd_hit | 90.96% | 90.36% | 89.55% | 221,027 | 106,249 | 22,365 | -1.75*10^10^ |
| no_cd_hit | 91.36% | 90.66% | 89.88% | 301,156 | 149,805 | 22,365 | -1.76*10^10^ |

**S2 Table. List of distribution of 12 top putative TFs among tissues.** p-value from the goodness-of-fit test

| TF | PF | PL | PM | PR | P-value |
| --- | --- | --- | --- | --- | --- |
| bZIP | 385 | 413 | 448 | 445 | 0.09897 |
| bHLH | 347 | 408 | 458 | 470 | 5.45*10^-5^ |
| NAC | 372 | 392 | 427 | 405 | 0.2609 |
| MYB_related | 291 | 355 | 331 | 345 | 0.06655 |
| ARF | 286 | 309 | 394 | 395 | 3.70*10^-6^ |
| C2H2 | 262 | 291 | 302 | 307 | 0.2417 |
| C3H | 253 | 288 | 292 | 297 | 0.2356 |
| MYB | 245 | 249 | 286 | 311 | 0.01228 |
| G2-like | 193 | 241 | 212 | 233 | 0.09438 |
| WRKY | 209 | 215 | 263 | 249 | 0.03252 |
| HD-ZIP | 203 | 211 | 285 | 287 | 1.24*10^-5^ |
| ERF | 194 | 207 | 279 | 273 | 2.11*10^-5^ |
| Total | 3240 | 3579 | 3977 | 4017 | 2,20*10^-16^ |

**S3 Table. List of genes that are used for salt-resistant analysis.** They all have been tested as having a salt stress response in certain plants.

| Gene | Product | Organism | Uniprot ID |
| --- | --- | --- | --- |
| *NHX7* | Sodium/hydrogen exchanger 7 (SOS1) | *A. thaliana* | Q9LKW9 |
| *CIPK24* | CBL-interacting serine/threonine-protein kinase 24 (SOS2) | *O. sativa subsp. Japonic* | Q69Q47 |
| *CNBL4* | Calcineurin B-like protein 4 (SOS3) | *O. sativa subsp. Japonic* | Q75KU4 |
| *PK* | Pyridoxal kinase (SOS4) | *A. thaliana* | Q8W1X2 |
| *FLA4* | Fasciclin-like arabinogalactan protein 4 (SOS5) | *A. thaliana* | Q9SNC3 |
| *SALT* | Salt-stress-induced protein | *O. sativa subsp. indica* | A2WPN7 |
