# Supplementary figures and images for: Optimization and application of non-native Phragmites australis transcriptome assemblies
Source: PLoS One. 2023 Jan 23;18(1):e0280354. doi: 10.1371/journal.pone.0280354 (PMC9870158; doi:10.1371/journal.pone.0280354)

**a. Trinity**

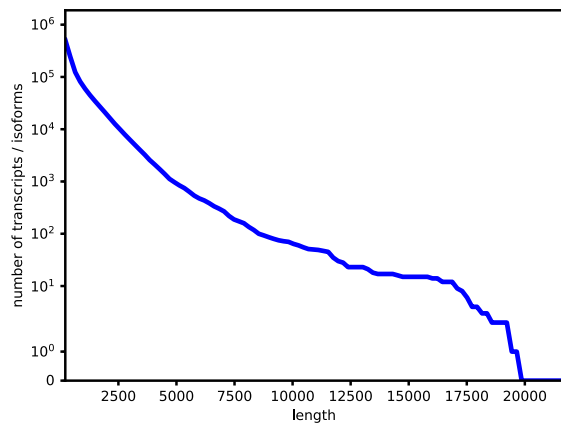

**b. Trinity\_GG**

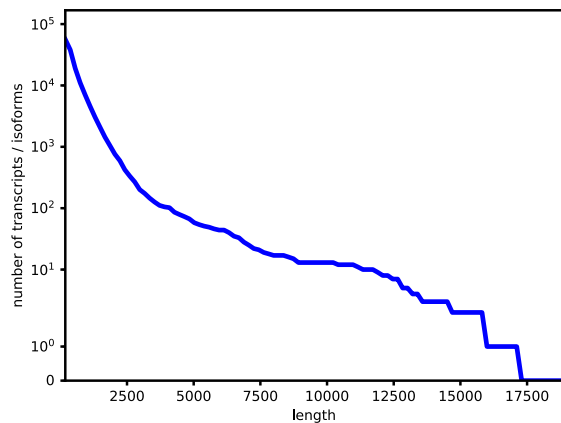

**c. GapClosed**

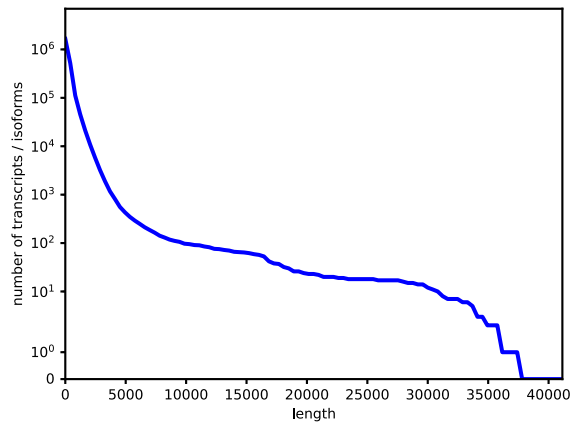

**d. Transabyss**

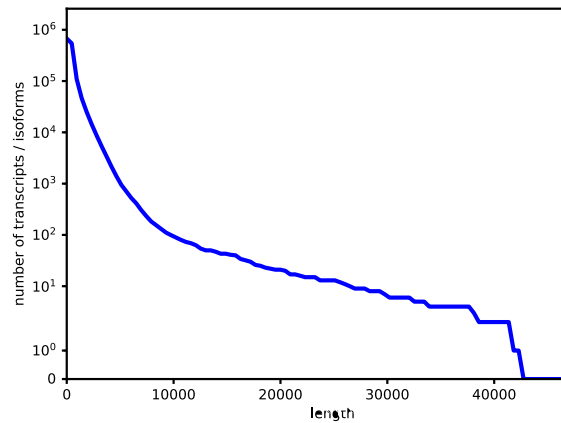

**e. Shannon**

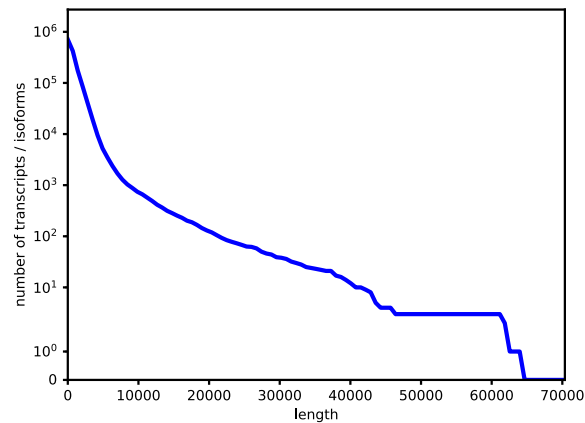

**f. cd\_hit**

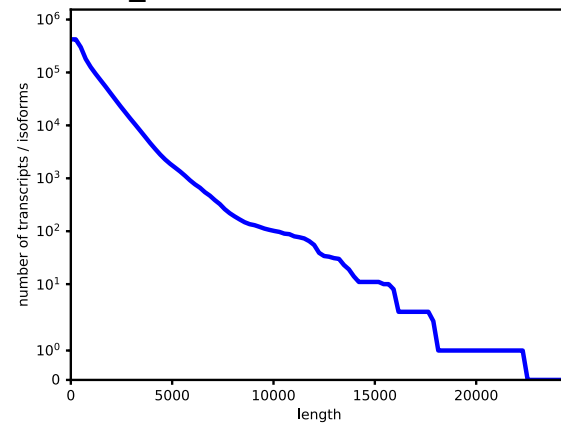

**g. no\_cd\_hit**

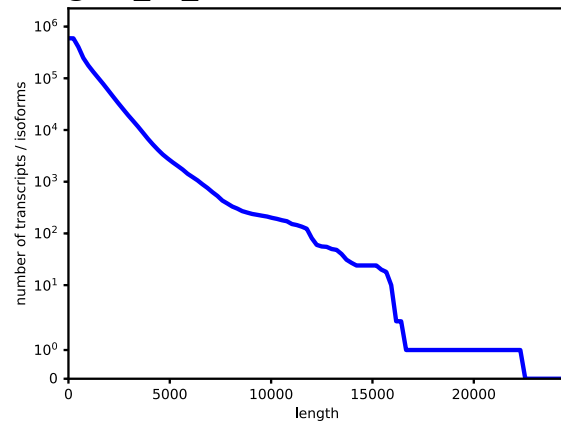

Supplement: S1 Fig — (PDF) [file pone.0280354.s002.pdf]

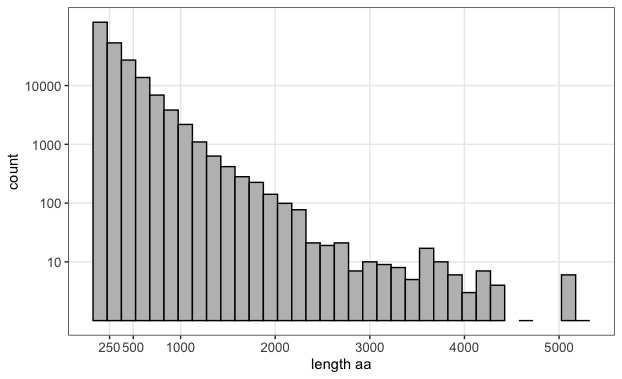

Supplement: S2 Fig — (PNG) [file pone.0280354.s003.png]

a. Sample correlation based on transcripts' expressions

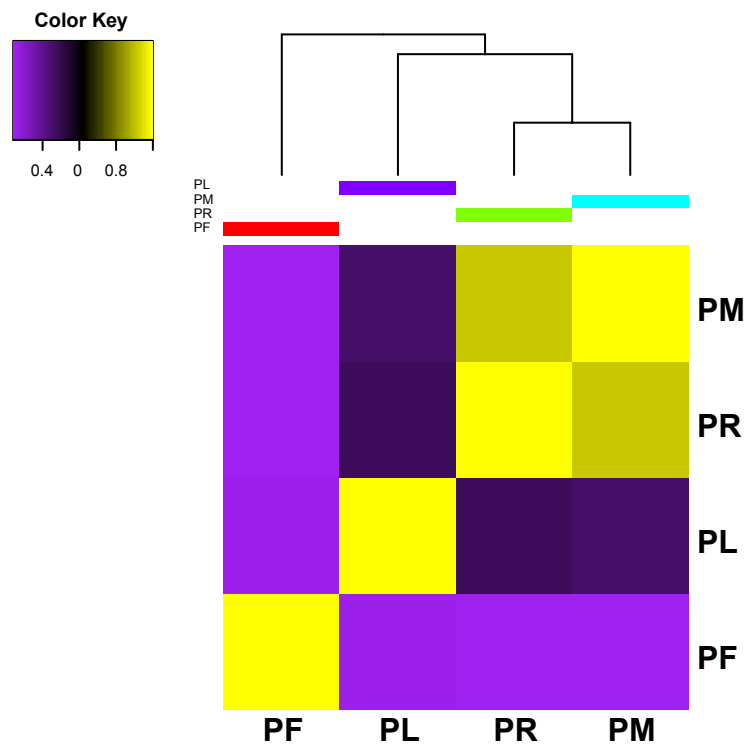

b. PCA analysis on sample correlation

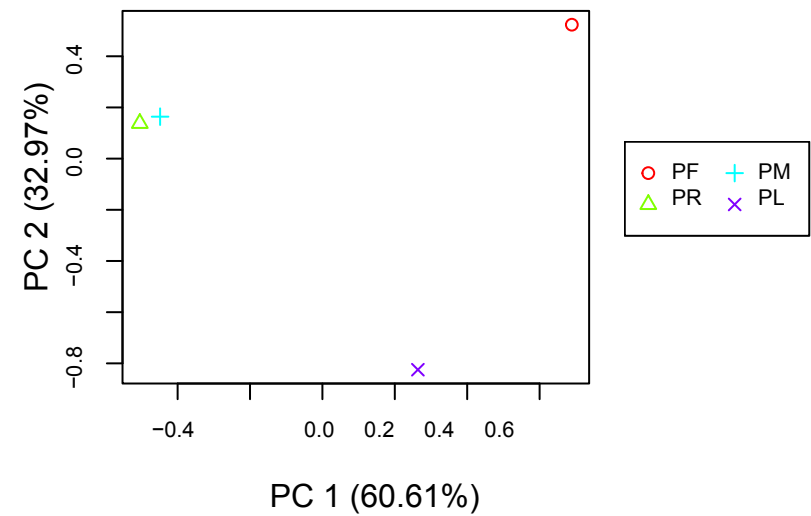

Supplement: S3 Fig — They are all from the NR blastx annotation. PL: leaf, PF: inflorescence, PM: shoot meristem, and PR: rhizome. (PDF) [file pone.0280354.s004.pdf]

a.

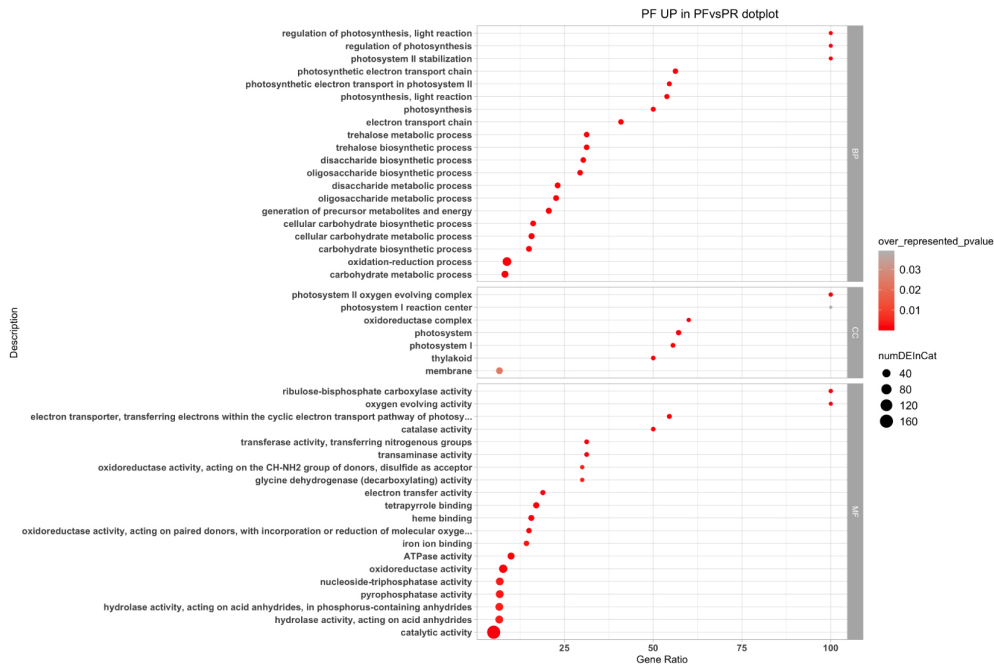

b.

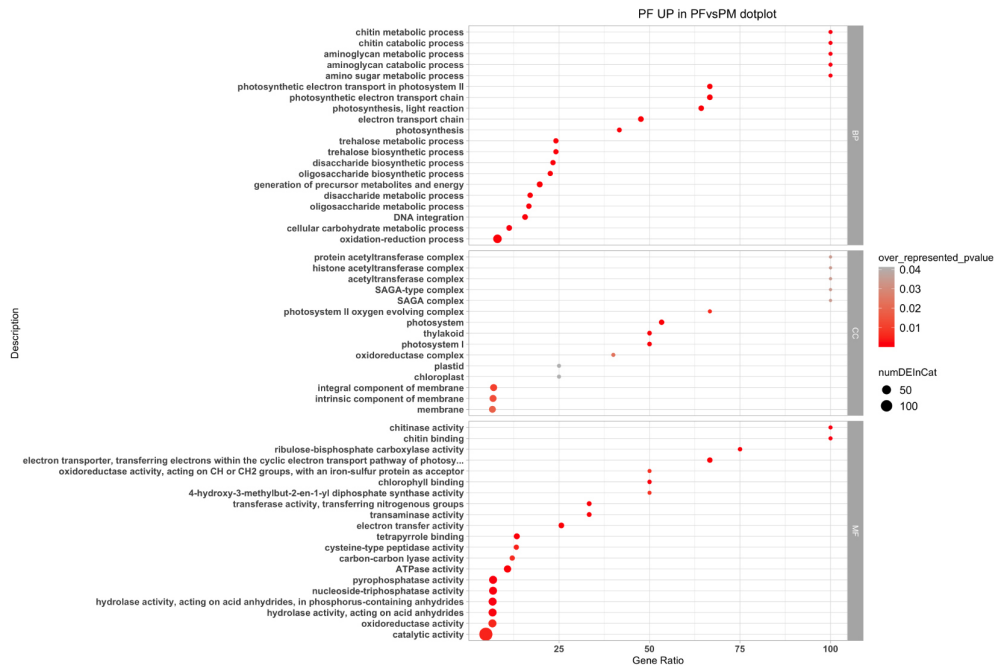

c.

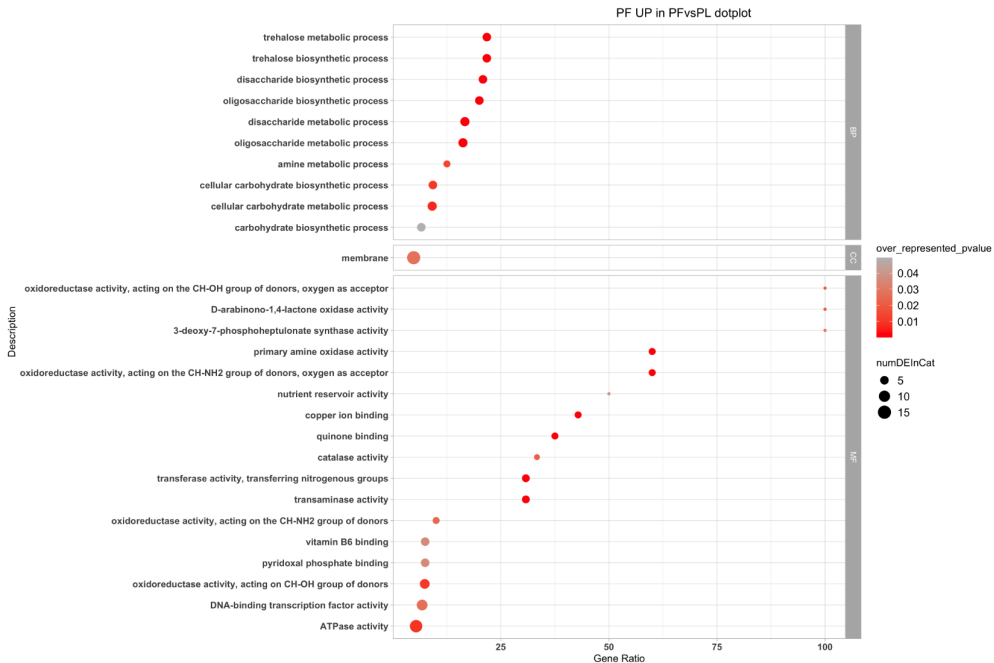

d.

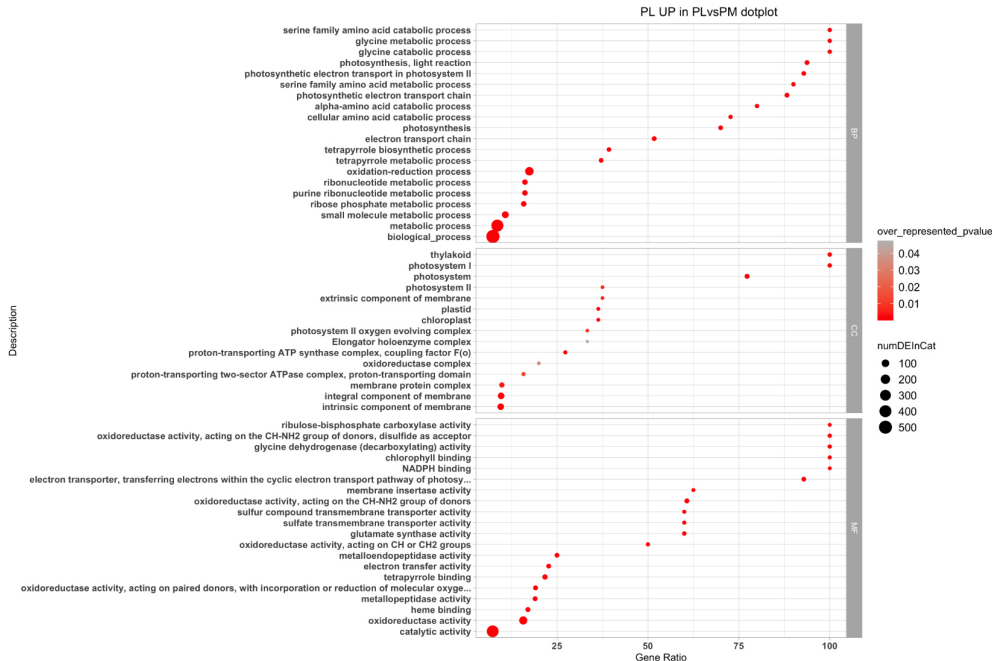

e.

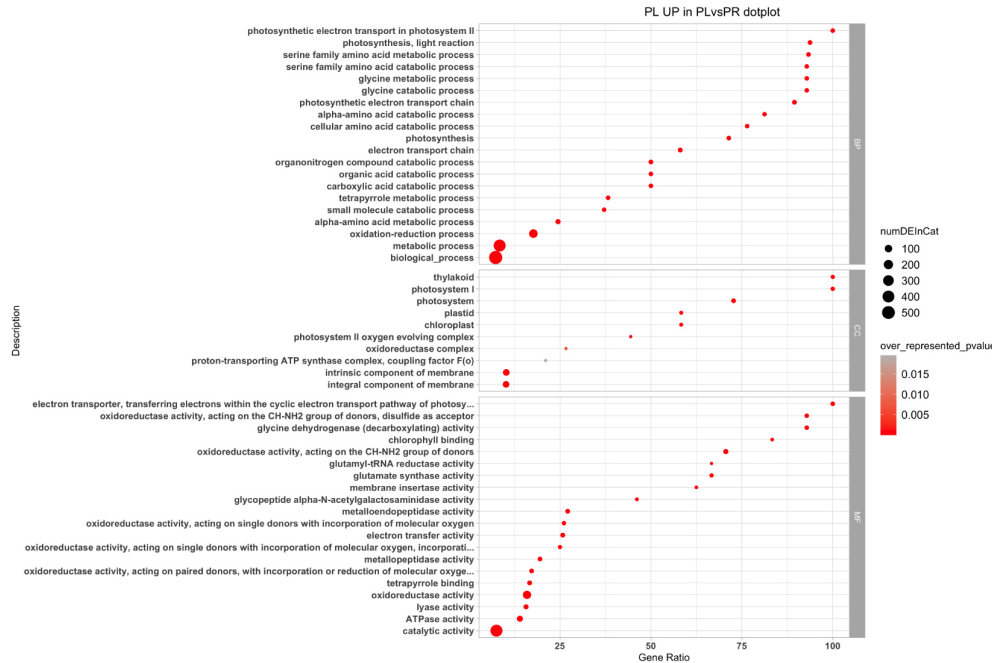

f.

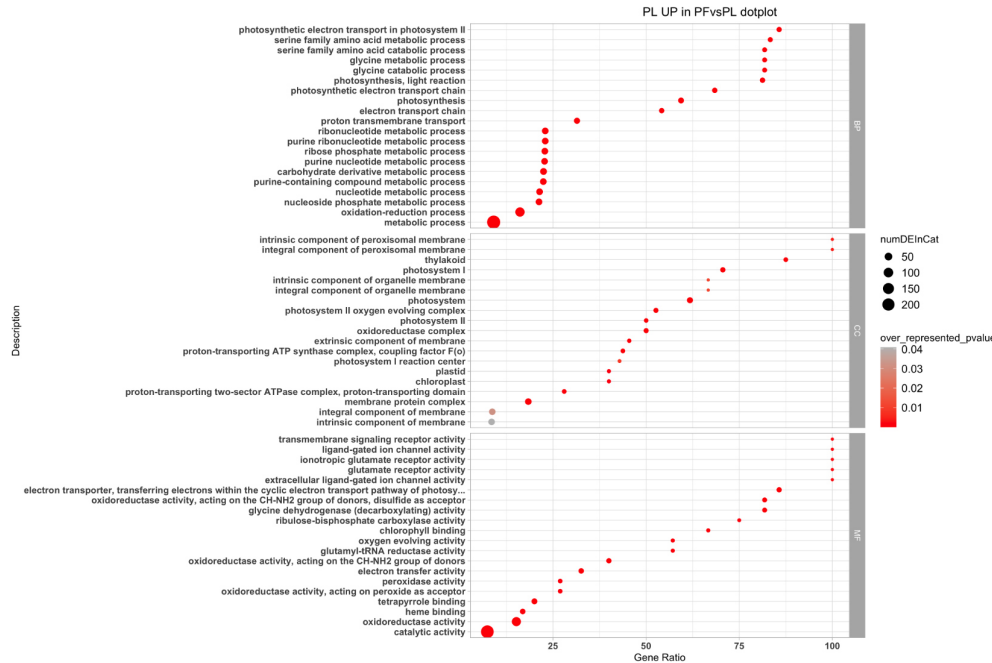

g.

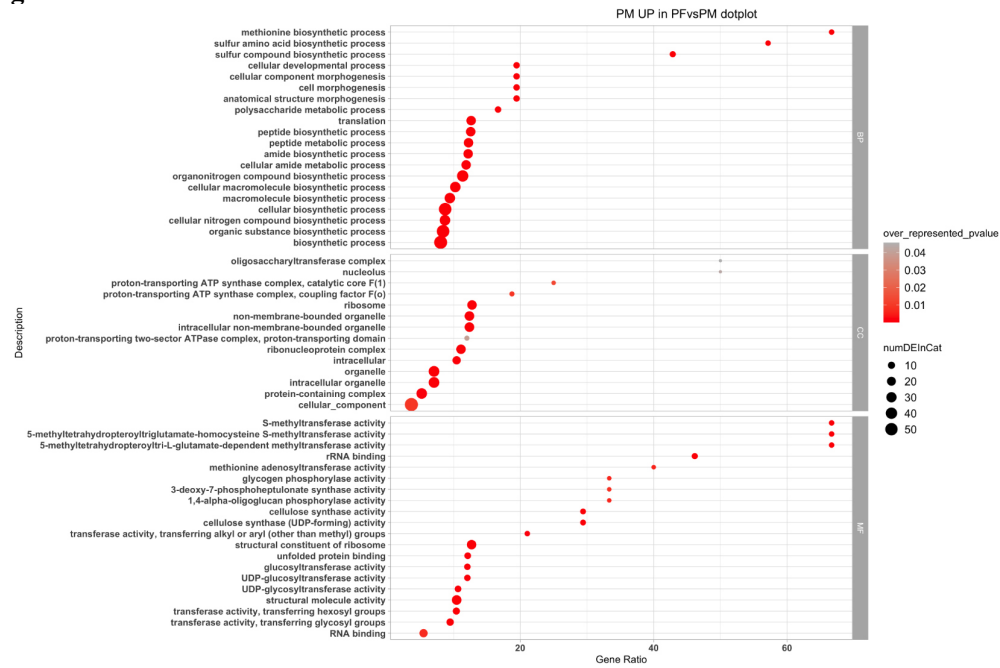

h.

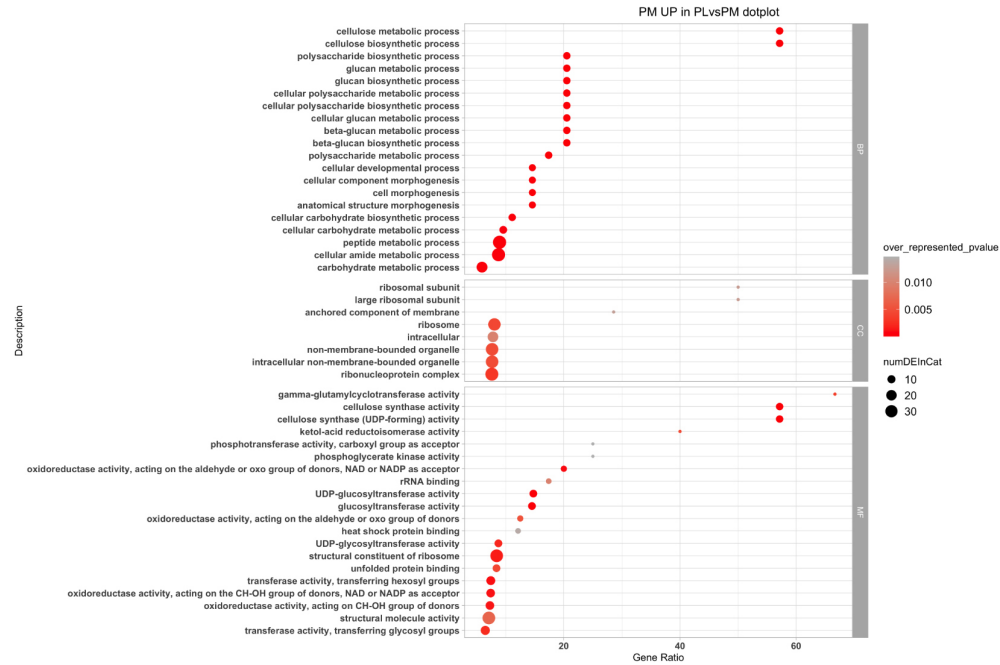

i.

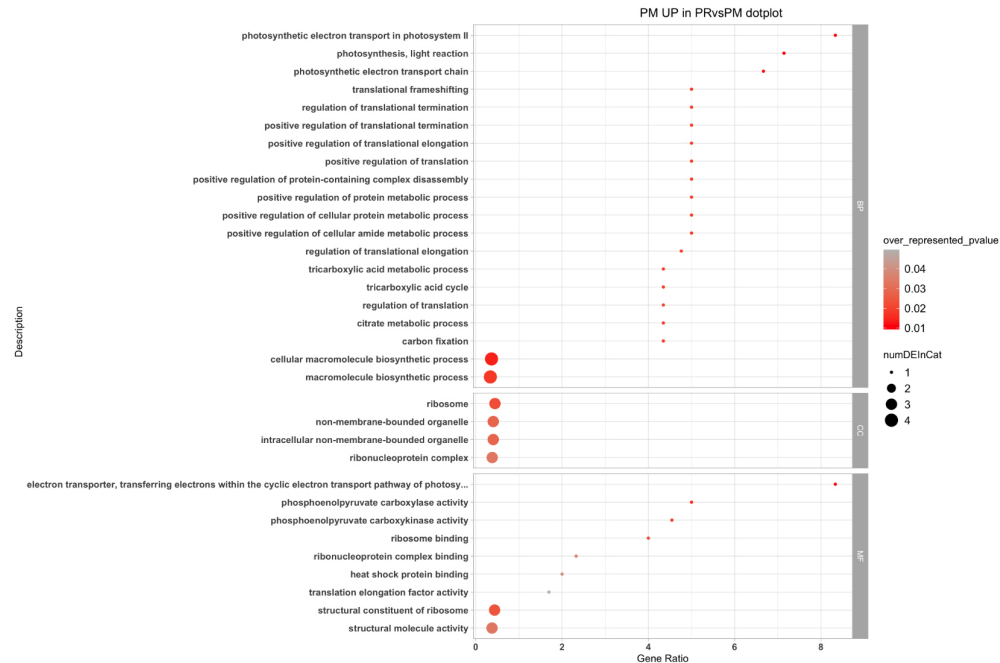

j.

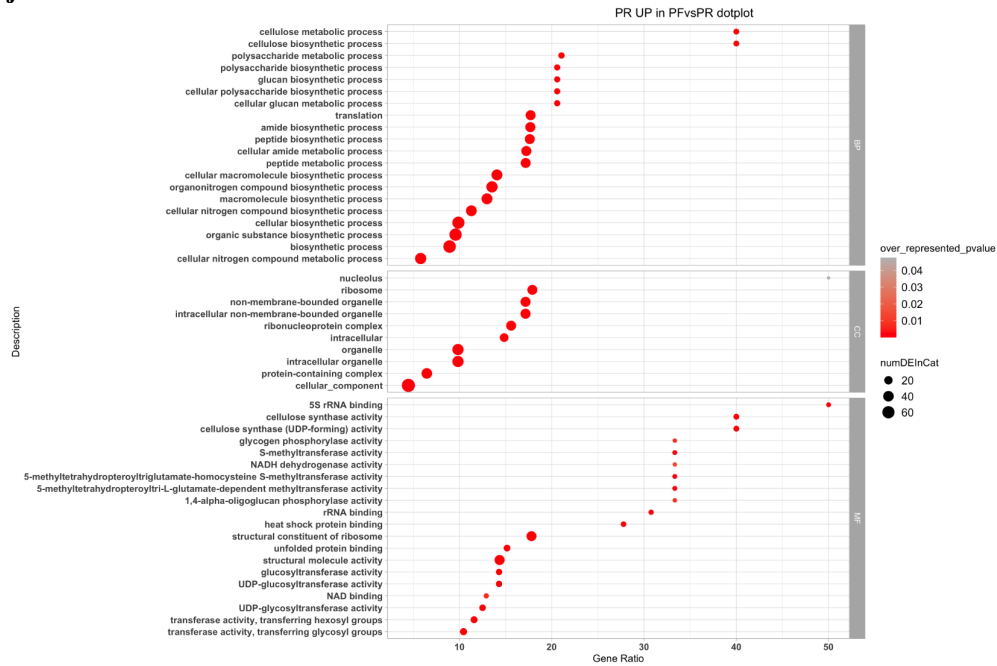

k.

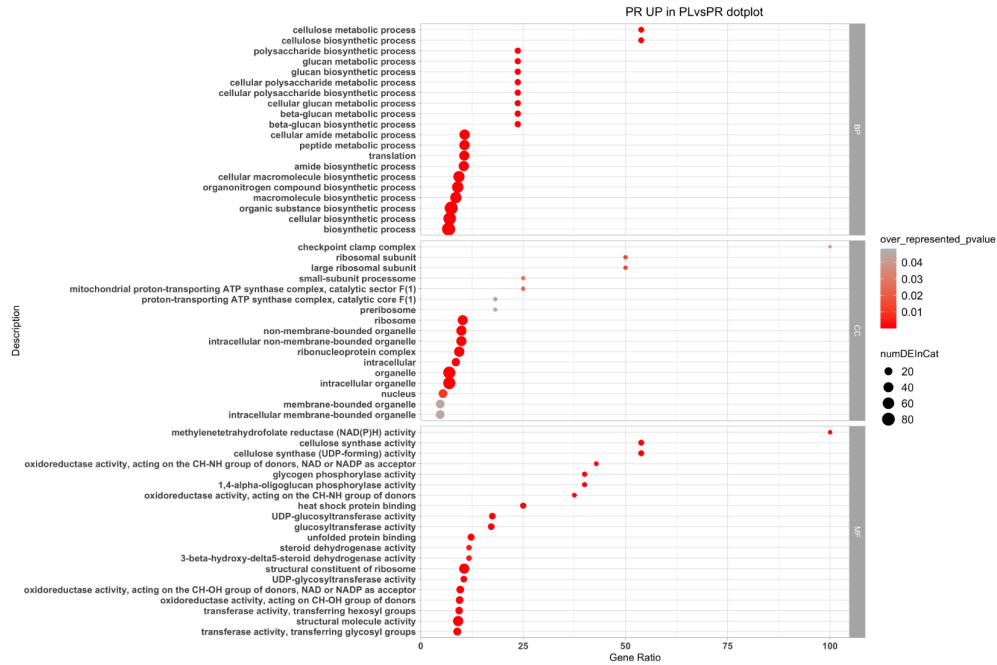

l.

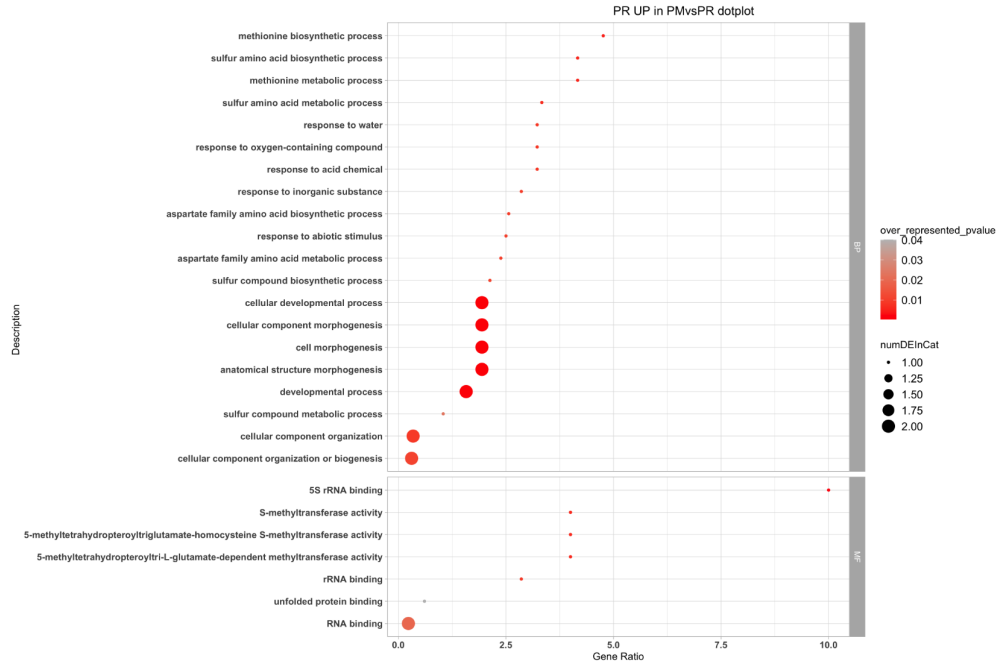

Supplement: S5 Fig — Six main clusters (a to f) having similar expression patterns in four tissues were extracted by Trinity script define_clusters_by_cutting_tree.pl. The number of transcripts in each cluster is listed after the cluster name in the title. (PDF) [file pone.0280354.s006.pdf]

a.

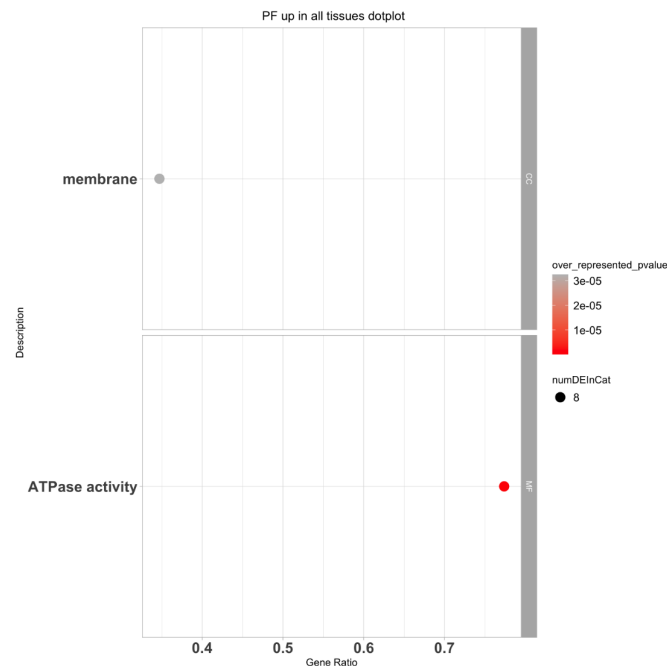

b.

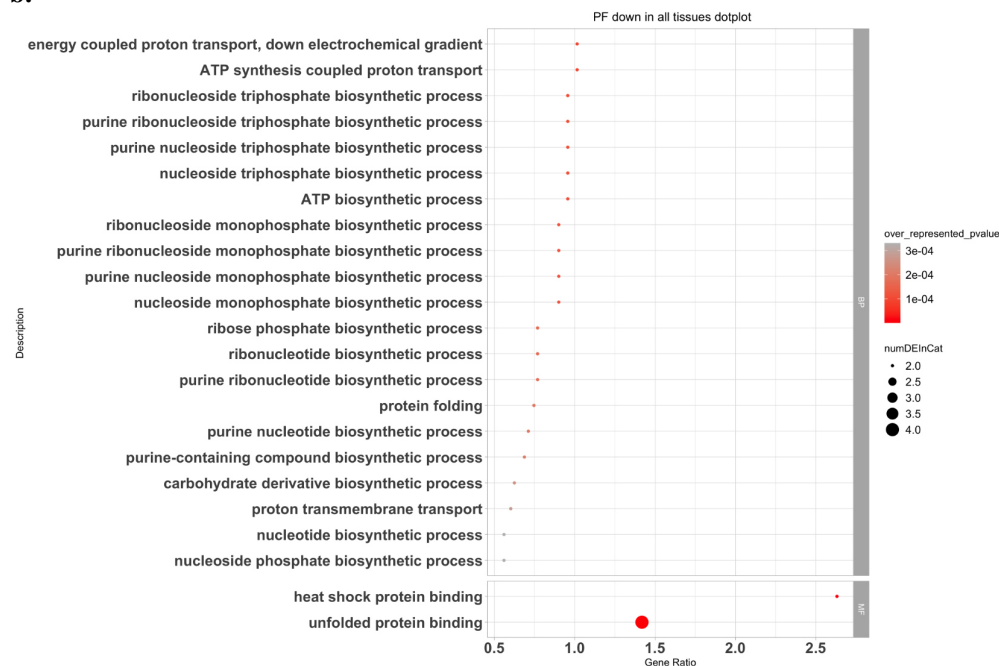

c.

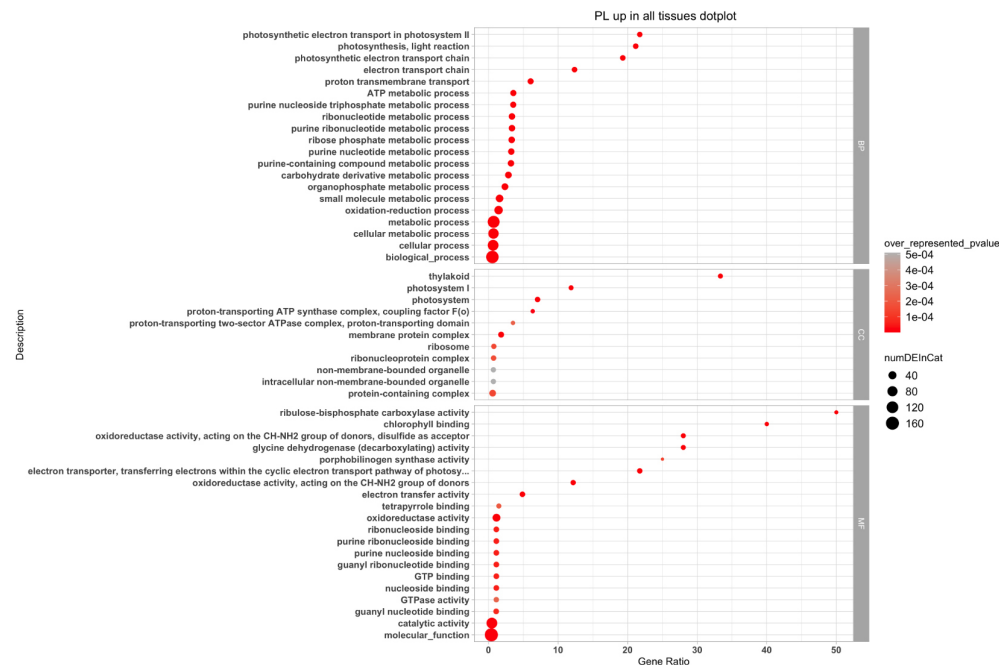

d.

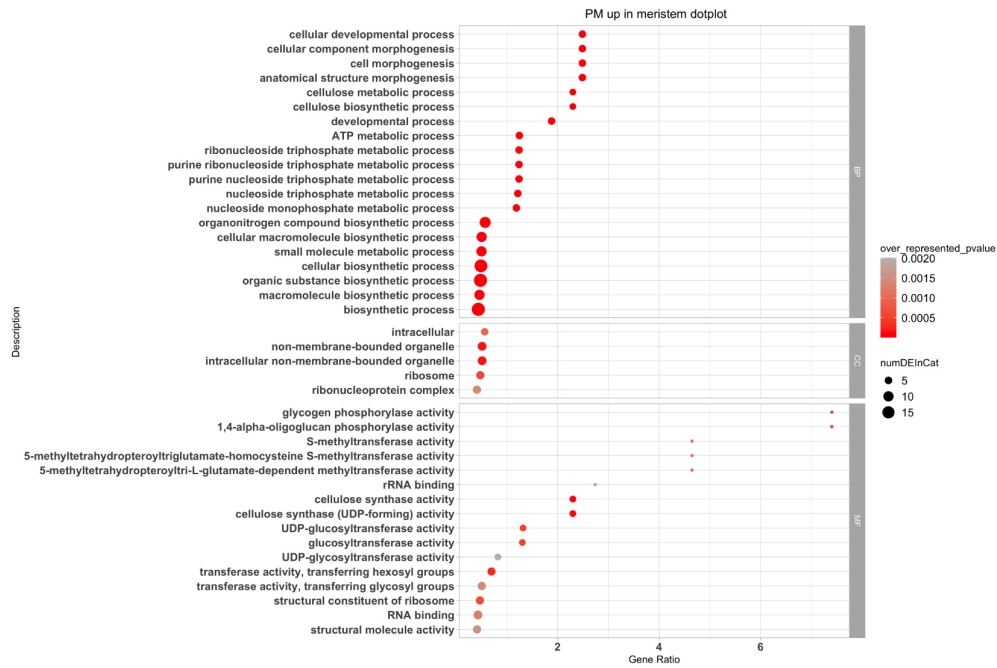

e.

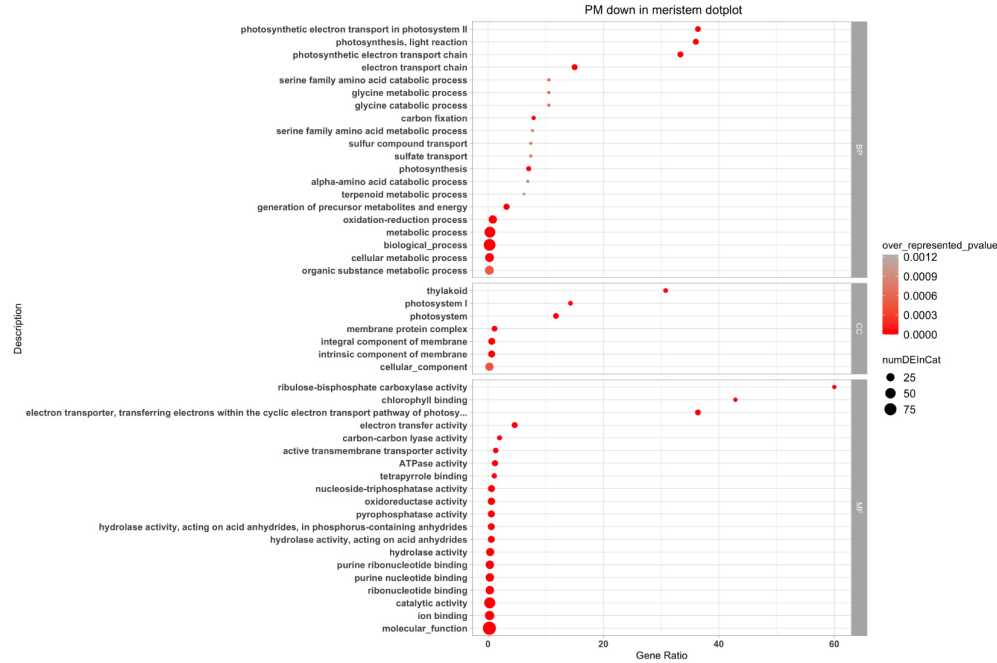

f.

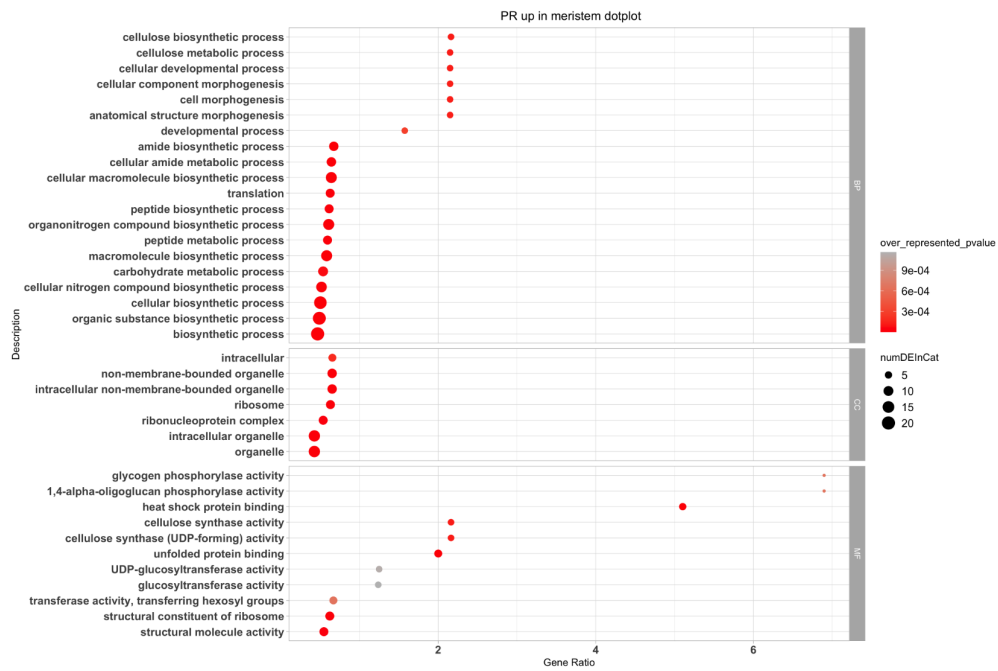

g.

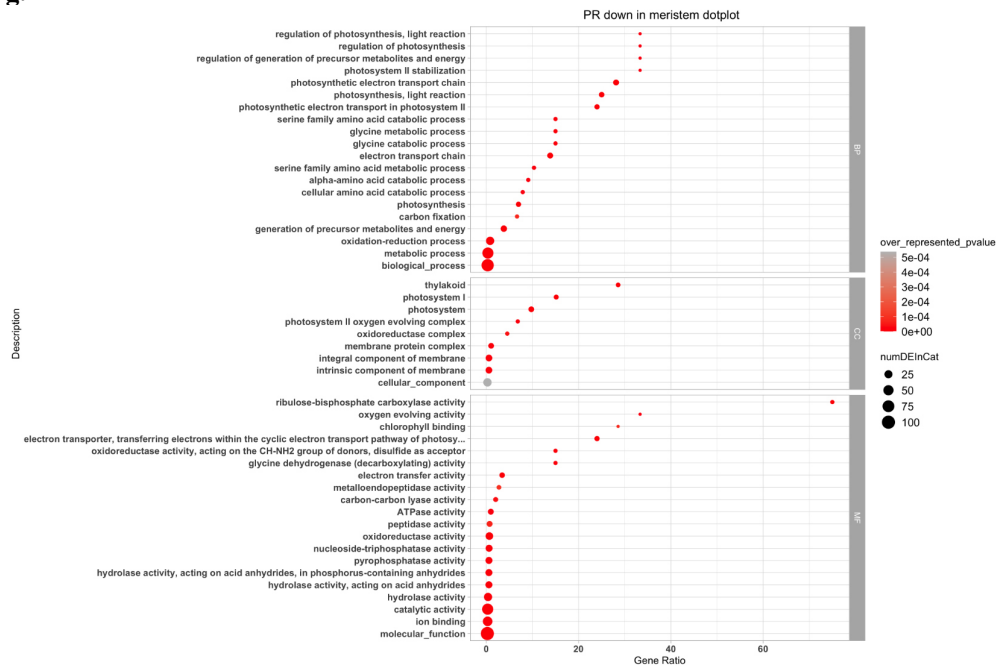

Supplement: S6 Fig — a. GO enrichment analysis on up-regulated DETs in PF between PF and PR; b. GO enrichment analysis on up-regulated DETs in PF between PF and PM; c. GO enrichment analysis on up-regulated DETs in PF between PF and PL; d. GO enrichment analysis on up-regulated DETs in PL between PL and PM; e. GO enrichment analysis on up-regulated DETs in PL between PL and PR; f. GO enrichment analysis on up-regulated DETs in PL between PL and PF; g. GO enrichment analysis on up-regulated DETs in PM between PM and PF; h. GO enrichment analysis on up-regulated DETs in PM between PM and PL; i. GO enrichment analysis on up-regulated DETs in PM between PM and PR; j. GO enrichment analysis on up-regulated DETs in PR between PR and PF; k. GO enrichment analysis on up-regulated DETs in PR between PR and PL; l. GO enrichment analysis on up-regulated DETs in PR between PR and PM. The size of the circle indicates the transcript number. Each different color indicates a different FDR value. (PDF) [file pone.0280354.s007.pdf]

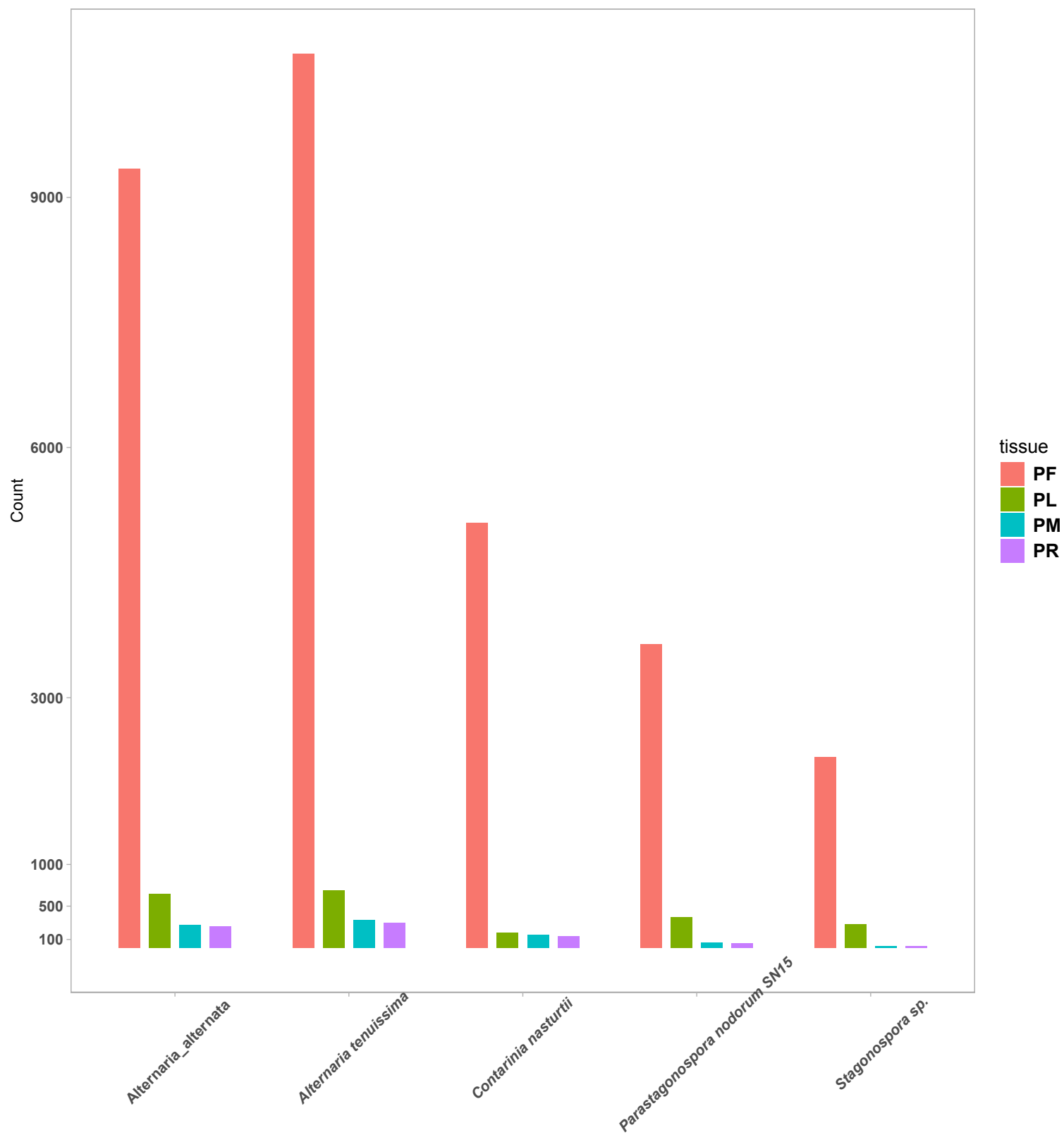

Supplement: S7 Fig — a. GO enrichment analysis on up-regulated DETs specific in PF; b. GO enrichment analysis on down-regulated DETs specific in PF; c. GO enrichment analysis on up-regulated DETs specific in PL; d. GO enrichment analysis on up-regulated DETs specific in PM; e. GO enrichment analysis on down-regulated DETs specific in PM; f. GO enrichment analysis on up-regulated DETs specific in PR; g. GO enrichment analysis on down-regulated DETs specific in PR. The size of the circle indicates the transcript number. Each different color indicates a different FDR value. (PDF) [file pone.0280354.s008.pdf]
