# Supplementary material for: Optimization and application of non-native Phragmites australis transcriptome assemblies
Source: PLoS One. 2023 Jan 23;18(1):e0280354. doi: 10.1371/journal.pone.0280354 (PMC9870158; doi:10.1371/journal.pone.0280354)
Supplement: S4 Fig — a. Heatmap shows the samples’ correlation based on the transcript’s expression matrix. Yellow depicts high value and blue depicts lower value; b. PCA plot based on the transcript’s expression matrix. PL: leaf, PF: inflorescence, PM: shoot meristem, and PR: rhizome. (PDF) [file pone.0280354.s005.pdf]

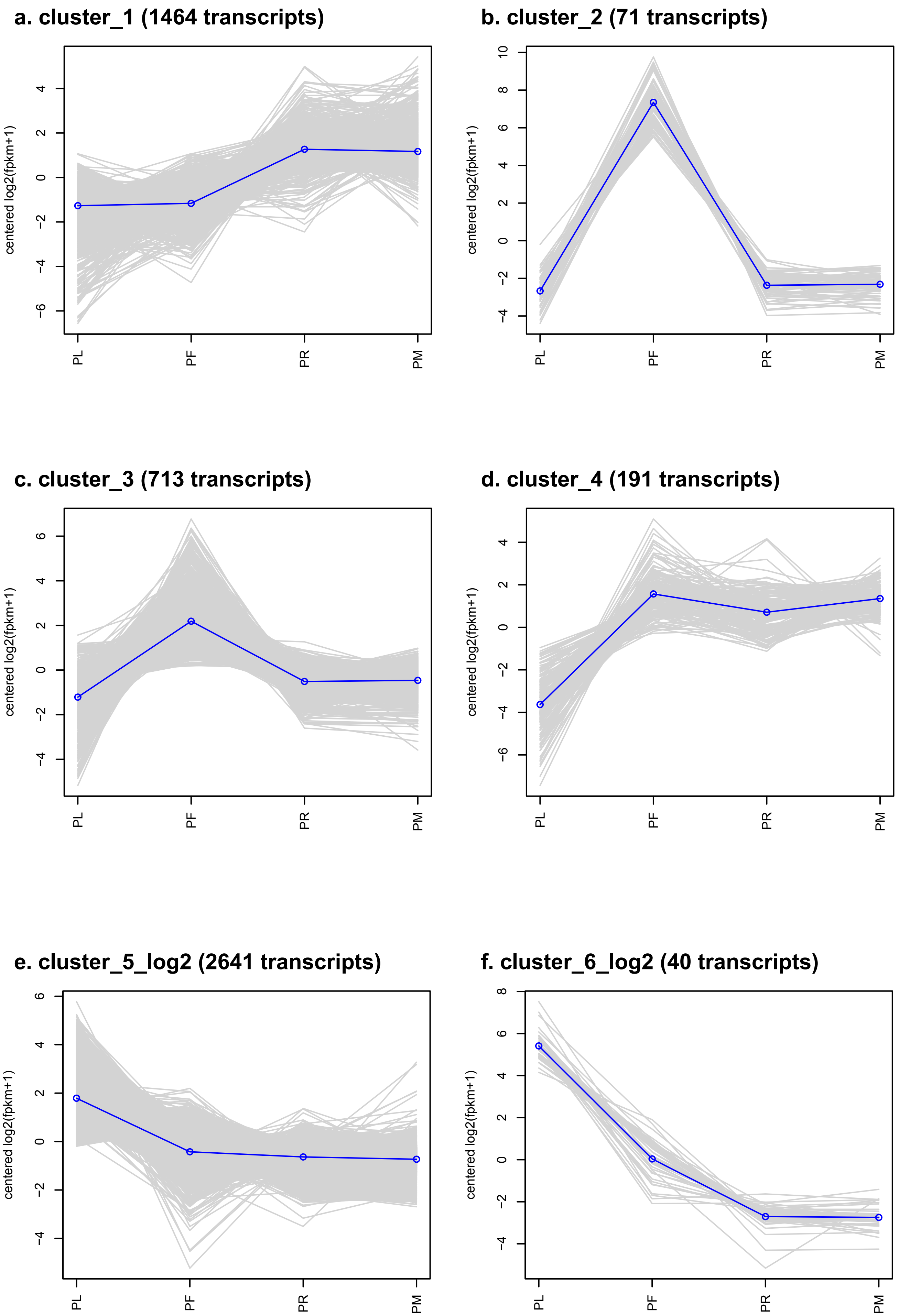
a. cluster_1 (1464 transcripts)
b. cluster_2 (71 transcripts)
c. cluster_3 (713 transcripts)
d. cluster_4 (191 transcripts)
e. cluster_5_log2 (2641 transcripts)
f. cluster_6_log2 (40 transcripts)
centered log2(fpkm+1)
PL
PF
PR
PM
